# Supplementary material for: A nontyphoidal Salmonella serovar domestication accompanying enhanced niche adaptation
Source: EMBO Mol Med. 2022 Sep 29;14(11):e16366. doi: 10.15252/emmm.202216366 (PMC9641423; doi:10.15252/emmm.202216366)
Supplement: Supplementary file 1 — Appendix [file EMMM-14-e16366-s009.pdf]

Appendix Supplementary Methods

A non-typhoidal *Salmonella* serovar domestication accompanying enhanced niche adaptation

Table of content

**Table**	**Page**
Appendix Table S1. Conjugation frequency of *Salmonella* Livingstone isolates.	2
Appendix Figure S1. Growth curves of *S*. Livingstone isolates of 4 clades.	3
Appendix Figure S2. Survival of *C. elegans* challenged with *Salmonella* Livingstone.	4

Appendix Table S1. Conjugation frequency of *Salmonella* Livingstone isolates.

Name of isolates	Clade	Number of transconjugants (CFU)	Number of recipients (CFU)	Conjugation frequency
L12	C5-I-b	2.2×10^{6}	5.0×10^{6}	4.4×10^{-2}
L35	C5-I-b	1.4×10^{1}	3.0×10^{6}	4.7×10^{-6}
L41	C5-I-a	1.2×10^{2}	3.0×10^{6}	4.0×10^{-5}
L42	C5-I-a	1.6×10^{7}	8.0×10^{7}	2.0×10^{-2}
Lin-10	C5-I-a	2.0×10^{1}	1.0×10^{7}	2.0×10^{-6}

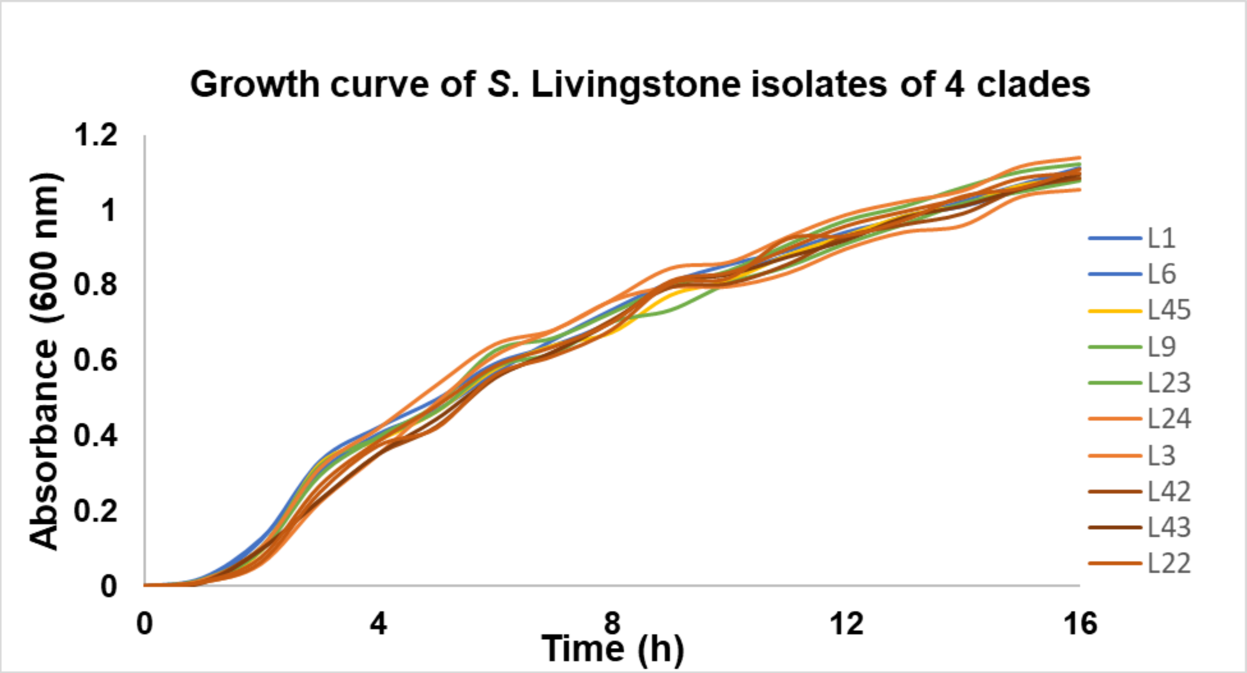

Appendix Figure S1. Growth curves of *S*. Livingstone isolates of 4 clades. The growth curves of the 10 isolates (Clade-1: strain L1 and L6; Clade-2: strain L45; Clade-3: strain L9 and L23; Clade-5: Clade-5-I-a strain L22, Clade-5-I-b strain L42 and L43, Clade-5-I-c strain L3 and L24) from 4 different clades were measured.

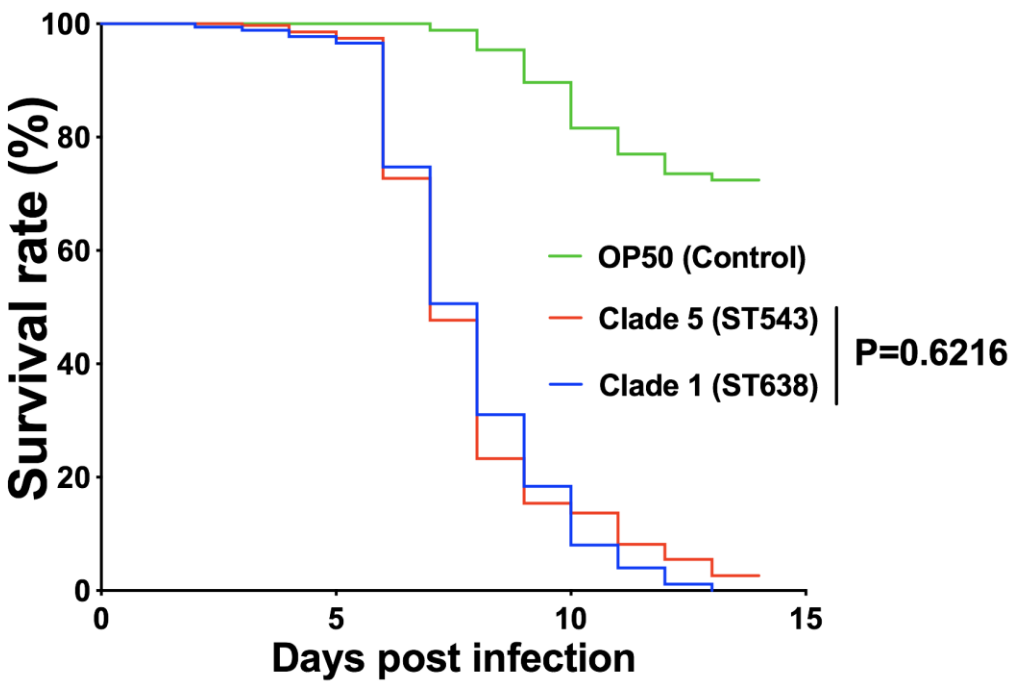

Appendix Figure S2. Survival of *C. elegans* challenged with *Salmonella* Livingstone. *C. elegans* was infected with isolates from Clade-1 and Clade-5 to evaluated their survival rates until day 14. The P value between two clades was calculated using Log-rank (Mantel-Cox, chi-square) test.
