# Supplementary material for: A nontyphoidal Salmonella serovar domestication accompanying enhanced niche adaptation
Source: EMBO Mol Med. 2022 Sep 29;14(11):e16366. doi: 10.15252/emmm.202216366 (PMC9641423; doi:10.15252/emmm.202216366)
Supplement: Supplementary file 2 — Expanded View Figures PDF [file EMMM-14-e16366-s004.pdf]

Expanded View Figures

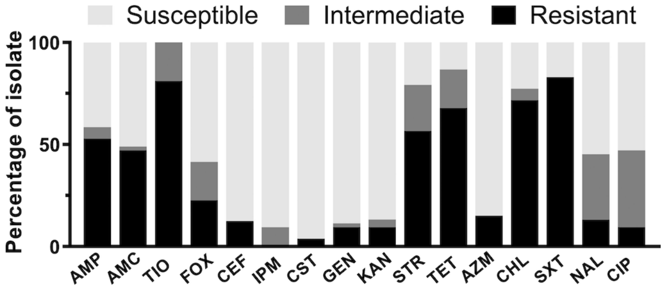

Figure EV1. Antimicrobial susceptibility testing.

A total of 53 SL isolates were tested for susceptibility to 16 antimicrobials in aerobic conditions, i.e. ampicillin (AMP), amoxicillin-clavulanic acid (AMC), ceftiofur (-TIO), cefoxitin (FOX), imipenem (IPM), gentamicin (-GEN), kanamycin (KAN), streptomycin (STR), tetracycline (TET), ciprofloxacin (CIP), nalidixic acid (NAL), trimethoprim-sulfamethoxazole (SXT), colistin (CST), azithromycin (AZM), ceftriaxone (CRO) and chloramphenicol (CHL). Cutoffs are listed in the source data file.

Source data are available online for this figure.

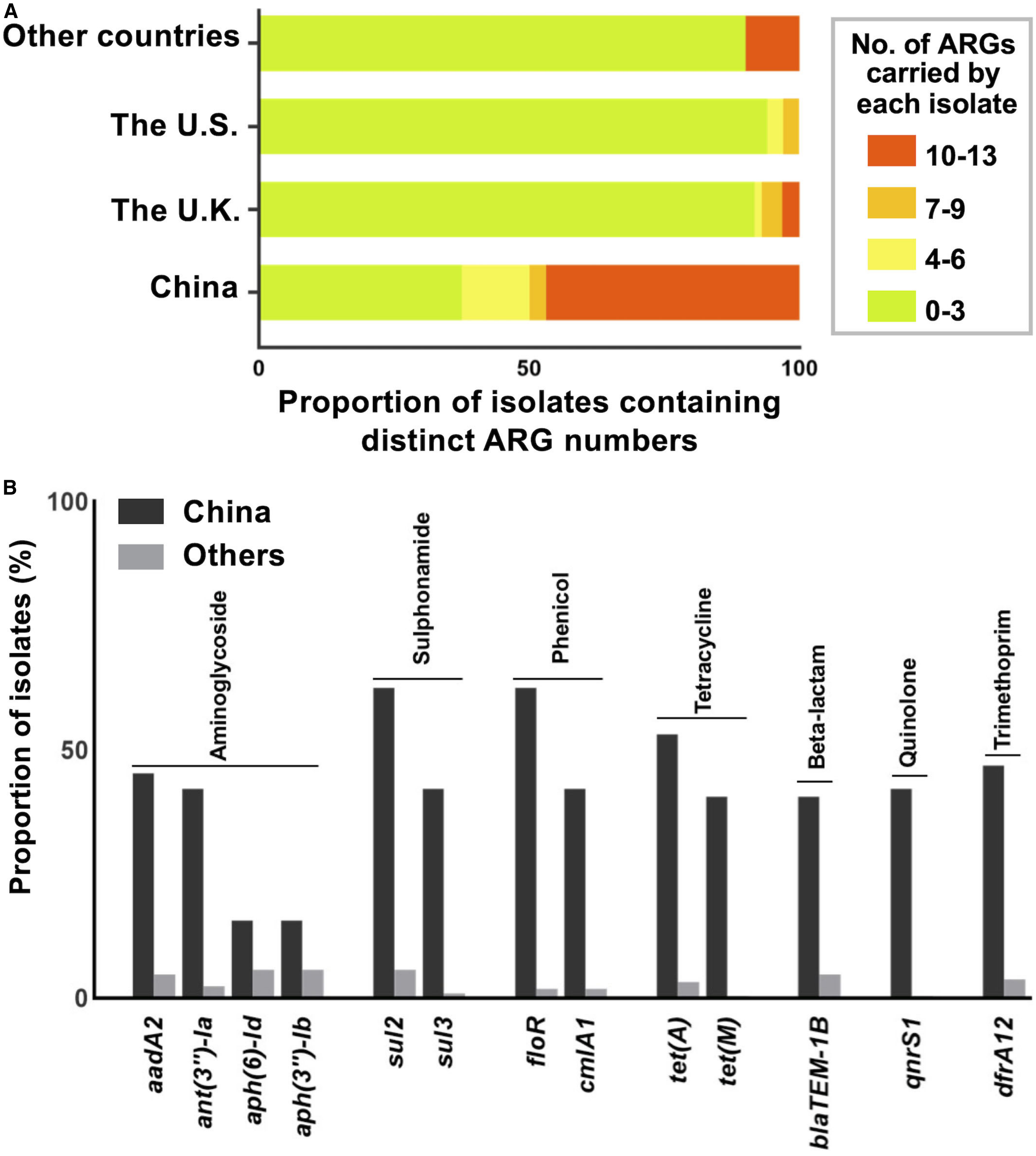

Figure EV2. Distribution of ARGs in SL isolates from China and other countries.

A Comparison of proportion of isolates containing distinct numbers of the antimicrobial-resistant gene from China and other countries.

B Proportion of ARGs in isolates from China and other countries. A total of 274 isolates were included in the analyses: 156 strains from the U.K.; 64 strains from China; 34 strains from the U.S.; 20 strains from other countries.

Source data are available online for this figure.

©2022 The Authors

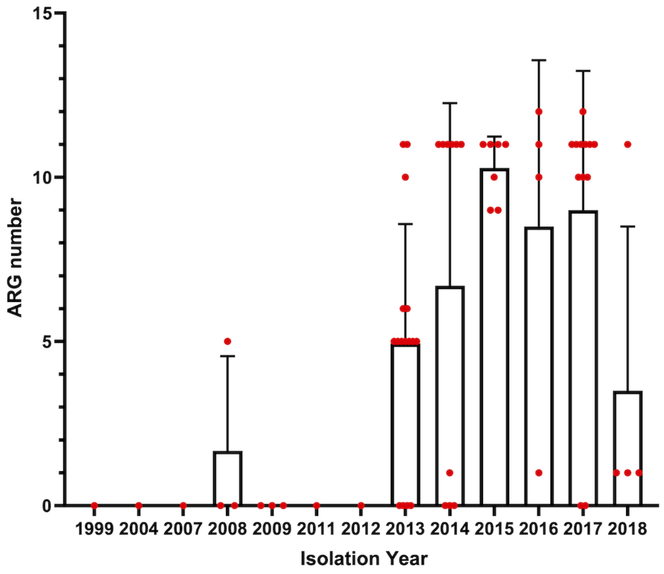

Figure EV3. The number of antimicrobial-resistant genes in isolates from distinct years.

The number of ARGs in all Chinese isolate were plotted against the isolation years. Red dots indicate the number of ARGs of each isolate. Data are presented as mean ± SD.

Source data are available online for this figure.

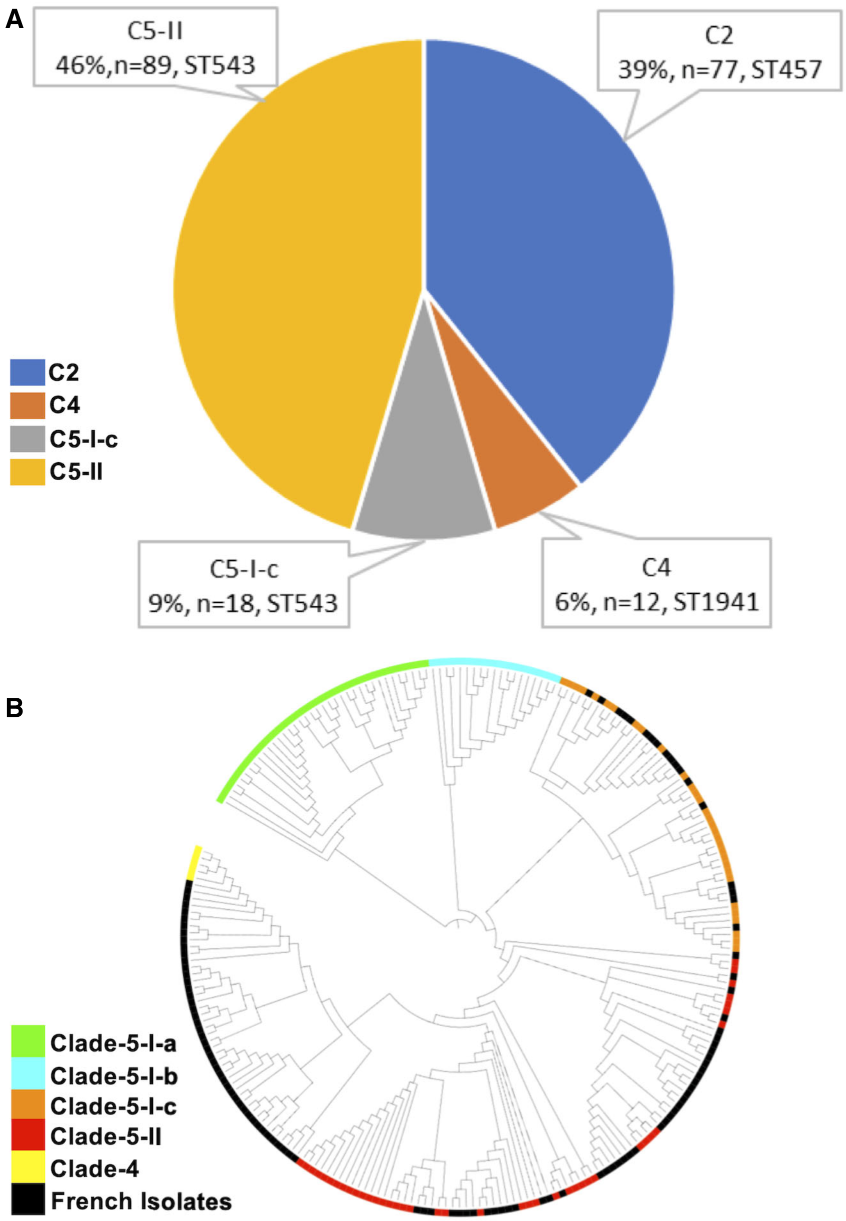

Figure EV4. Clade composition and phylogenetic tree of French isolates.

A The proportion of French isolates in each phylogenetic clade. The French isolates (*n* = 196) were from C2, C4, C5-I-c and C5-II.

B Phylogenetic relatedness of French and Chinese ST543 isolates. The French (*n* = 107) and Chinese ST543 isolates (*n* = 93) were used to construct a core-genome maximum-likelihood phylogenetic tree. The ring in different colours indicates the French isolates and distinct clades of Chinese isolates.

Source data are available online for this figure.

©2022 The Authors
