# Supplementary material for: A nontyphoidal Salmonella serovar domestication accompanying enhanced niche adaptation
Source: EMBO Mol Med. 2022 Sep 29;14(11):e16366. doi: 10.15252/emmm.202216366 (PMC9641423; doi:10.15252/emmm.202216366)
Supplement: Supplementary file 3 — Table EV1 [file EMMM-14-e16366-s006.docx]

**Table EV1. Clinical investigations of cases caused by invasive *Salmonella* Livingstone.**

| Strain | Isolation  Host | Age | Province (Isolation Year) | Isolation  Source | Failure Antibiotic Treatment | Successful Antibiotic Treatment | Phylogenetic clade | Antimicrobial-resistant profile^a^ |
| --- | --- | --- | --- | --- | --- | --- | --- | --- |
| L42 | Human  (Patient#1) | 4 months | Shanghai  (2016) | Blood | Ceftriaxone  (2 days), Azithromycin (3 days) | Imipenem (full course) | C5-I-b | AMP-AMC-TIO-FOX-CRO-GEN-KAN-STR-TET-AZM-CHL-SXT |
| L44 | Human  (Patient#2) | 8 months | Fujian  (2016) | Blood | Ceftriaxone  (3 days), Azithromycin (3 days) | Imipenem  (full course) | C5-I-a | AMP-AMC-TIO-STR-TET-AZM-CHL-SXT |
| L43 | Human  (Patient#3) | 51 years | Shanghai  (2016) | Blood | Ceftriaxone and Ciprofloxacin (5 days) | Colistin  (full course) | C5-I-b | AMP-AMC-TIO-FOX-CRO-GEN-KAN-STR-TET-AZM-CHL-SXT |
| 18B003 | Human  (Patient#4) | 45 years | Zhejiang  (2019) | Blood | Azithromycin (5 days) | Ceftriaxone  (full course) | C5-I-a | AMP-AMC-TIO-STR-TET-CHL-SXT-NAL |
| L39 | Human  (Patient#5) | 1 year | Chongqing  (2015) | Synovial fluid |  | Ceftriaxone  (full course) | C5-I-a | STR-TET-CHL-STX |

1. Abbreviations: ampicillin (AMP), amoxicillin-clavulanic acid (AMC), ceftiofur (TIO), cefoxitin (FOX), imipenem (IPM), gentamicin (GEN), kanamycin (KAN), streptomycin (STR), tetracycline (TET), colistin (CST), azithromycin (AZM), ceftriaxone (CRO), chloramphenicol (CHL), nalidixic acid (NAL), and trimethoprim-sulfamethoxazole (SXT).
