# Supplementary material for: A nontyphoidal Salmonella serovar domestication accompanying enhanced niche adaptation
Source: EMBO Mol Med. 2022 Sep 29;14(11):e16366. doi: 10.15252/emmm.202216366 (PMC9641423; doi:10.15252/emmm.202216366)
Supplement: Supplementary file 12 — PDF+ [file EMMM-14-e16366-s013.pdf]

Report

SOURCE DATA | TRANSPARENT PROCESS | OPEN ACCESS

EMBO Molecular Medicine

A nontyphoidal *Salmonella* serovar domestication accompanying enhanced niche adaptation

Yan Li[1,2,3,†], Lin Teng[2,†], Xuebin Xu[4,†], Xiaomeng Li[2], Xianqi Peng[2], Xiao Zhou[2], Jiaxin Du[2], Yanting Tang[2], Zhijie Jiang[2], Zining Wang[1,2], Chenghao Jia[1,2], Anja Müller[5], Corinna Kehrenberg[5], Haoqiu Wang[6], Beibei Wu[7], François-Xavier Weill[8] & Min Yue[1,2,3,9,*]

Abstract

Invasive nontyphoidal *Salmonella* (iNTS) causes extraintestinal infections with ~15% case fatality in many countries. However, the mechanism by which iNTS emerged in China remains unaddressed. We conducted clinical investigations of iNTS infection with recurrent treatment failure, caused by underreported *Salmonella enterica* serovar Livingstone (SL). Genomic epidemiology demonstrated five clades in the SL population and suggested that the international animal feed trade was a likely vehicle for their introduction into China, as evidenced by multiple independent transmission incidents. Importantly, isolates from Clade-5-I-a/b, predominant in China, showed an invasive nature in mice, chicken and zebrafish infection models. The antimicrobial susceptibility testing revealed most isolates (> 96%) in China are multidrug-resistant (MDR). Overall, we offer exploiting genomics in uncovering international transmission led by the animal feed trade and highlight an emerging hypervirulent clade with increased resistance to frontline antibiotics.

Keywords antimicrobial resistance; evolution; host adaptation; invasive nontyphoidal *Salmonella*; Livingstone

Subject Categories Evolution & Ecology; Microbiology, Virology & Host Pathogen Interaction

DOI 10.15252/emmm.202216366 | Received 24 May 2022 | Revised 9 September 2022 | Accepted 12 September 2022 | Published online 29 September 2022

EMBO Mol Med (2022) 14: e16366

Introduction

The intercontinental spread of infectious agents is mainly accelerated by anthropogenic activities or the consequences of human impact. Many of these activities, i.e. industrial animal farming and the application of antimicrobial growth promoters, can select for new variants, including hypervirulent and antimicrobial resistant (AMR) clones (Li *et al*, 2022). Indeed, many pathogens, e.g. *Salmonella*, could be selected in immunocompromised individuals or chronic carriers, with an enhancing host-fitness adaptation (Klemm *et al*, 2016). These variants are of particular importance due to their potential for increased transmissibility, enhanced virulence or reduced effectiveness of vaccines against them, and ultimately, for new waves of spread, as illustrated by SARS-CoV-2 variants. Understanding how a new pathogen or variant emerges and evolves has ultimate priority in global public health.

Invasive nontyphoidal *Salmonella* (iNTS) is linked with extraintestinal infections (Johnson *et al*, 2018). iNTS infections were increasingly documented in clinical settings worldwide, and recent global estimates show an average mortality rate of 15%, with a higher rate in Africa and Southern Asia (Marchello *et al*, 2022). iNTS infections, which commonly present as bloodstream infections, are significant disease burdens over the world, with 535,000 cases and 77,500 death annually (GBD 2017 Non-Typhoidal Salmonella Invasive Disease Collaborators, 2019). However, the exact burden of iNTS is largely lacking in China (Xu *et al*, 2020; Qiu *et al*, 2021; Hu *et al*, 2022).

Salmonella enterica serovar Livingstone (SL) has typically been expected to cause gastrointestinal diseases in industrial countries (Old *et al*, 1994; Guerin *et al*, 2004; Bouallègue-Godet *et al*, 2005). Before the 2000s, SL was rarely reported in human salmonellosis cases in China. Recently, increasing incidents of invasive infections caused by SL have been detected in Chinese hospitals. Here, we detected emerging SL variants with invasive features from Chinese patients and further projected the spatiotemporal transmission history in a global context, with a hallmark of host adaptation

1 Hainan Institute of Zhejiang University, Sanya, China
2 Institute of Preventive Veterinary Science & Department of Veterinary Medicine, Zhejiang University College of Animal Sciences, Hangzhou, China
3 Zhejiang Provincial Key Laboratory of Preventive Veterinary Medicine, Hangzhou, China
4 Shanghai Municipal Center for Disease Control and Prevention, Shanghai, China
5 Institute for Veterinary Food Science, Faculty of Veterinary Medicine, Justus-Liebig University Giessen, Giessen, Germany
6 Hangzhou Center for Disease Control and Prevention, Hangzhou, China
7 Zhejiang Province Center for Disease Control and Prevention, Hangzhou, China
8 Department of Global Health, Institut Pasteur, Paris, France
9 State Key Laboratory for Diagnosis and Treatment of Infectious Diseases, National Clinical Research Center for Infectious Diseases, National Medical Center for Infectious Diseases, The First Affiliated Hospital, College of Medicine, Zhejiang University, Hangzhou, China
*Corresponding author. Tel: +86 571 88982832; E-mail: myue@zju.edu.cn
[†]These authors contributed equally to this work

©2022 The Authors. Published under the terms of the CC BY 4.0 license.

(invasive clade) and AMR drift (acquisition of antimicrobial determinants) for about 20 years in China.

Results

Global population diversity of *Salmonella* Livingstone

Recently, unexpected cases of SL invasive infection were observed in clinical settings in mainland China (Table EV1). Notably, five intractable cases (extraintestinal infections) were associated with the failure of empiric antimicrobial therapy. The causative agents were confirmed as SL (L39, L42, L43, L44 and 18B003) by whole genome sequencing (WGS).

To investigate the genetic relationship of these problematic isolates, phylogenomic analyses were conducted using WGS for Chinese isolates, including 53 from this study and 11 from public domains, contextualised with 210 global isolates (Fig 1A; Dataset EV1). Two hundred seventy-four isolates were from different sources (human, animal and food) and different geographical regions, mainly the U.K., U.S. and China (Fig 1B; Dataset EV1). The maximum-likelihood phylogenetic tree based on 85,853 core-genome SNPs revealed that global isolates formed five major clades. The C-1, C-2, C-3 and C-5 consisted of ST638, ST475, ST2247 and ST543 isolates, respectively, while the C-4 comprised isolates of several STs, including ST1941 (n = 28), ST2375 (n = 1) and ST3986 (n = 1) (Fig 1A). Notably, 5 iNTS strains belong to ST543 and C-5.

The population structure of SL was correlated with the geographical locations where they were sampled (Fig 1B). C-1 and C-3 comprised isolates mostly from the U.S., while the isolates from the U.K. were distributed in C-2, C-4 and C-5. The Chinese isolates fall predominantly into C-5 (n = 57), with several isolates sporadically spread in C-1 (n = 4), C-2 (n = 1) and C-4 (n = 2). Interestingly, 47% of SL isolates in C-1 were from poultry (8/38) and animal feeds (10/38), mainly from the U.S. and U.K. The Chinese clinical isolates from C-1 (L6 and L36) were closely related to the strains from poultry, food, livestock and humans from the above two countries. The only Chinese isolate from C-2 (L45) was from patient stool, while another Chinese isolate of C-4 (L23) was from imported bovine meat and bone meal from Argentina. Notably, multiple Chinese SL isolates in C-1 and C-5 were identified in imported animal feed, i.e., fish meal and rapeseed meal, from the U.S., U.K. and Pakistan (Dataset EV1), indicating that international trade of animal feed is a source to introduce this bacterium into China. Besides, C-5 contained Chinese isolates from the blood and stools of patients with food poisoning and samples from different stages along the meat production chain, including animal feeds, animal carcasses at slaughterhouses and animal products in markets. The presence of phylogenetically related isolates along the meat production chain suggests farm-to-fork dissemination of SL, with animal feed as a critical vehicle being the initial source. Altogether these data suggest

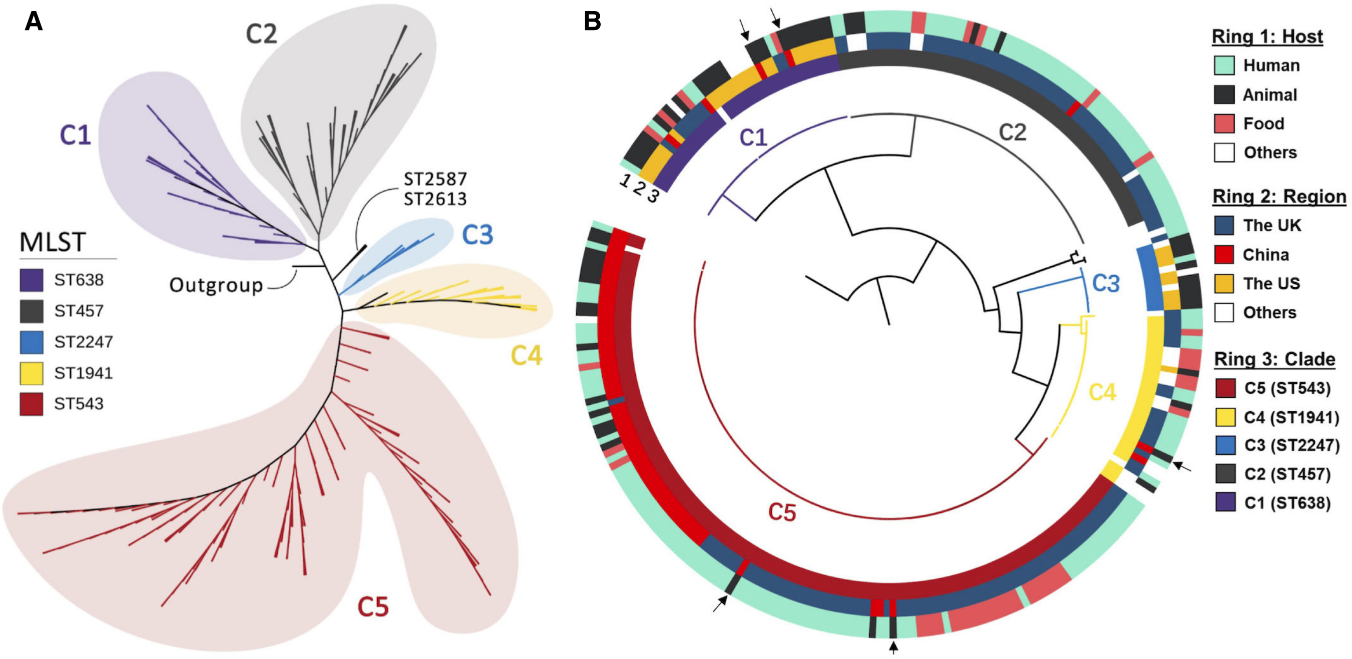

Figure 1. Maximum-likelihood phylogenetic tree of global SL isolates.

A Phylogenetic tree (unrooted) showing five major clades of global SL isolates. The phylogenetic trees were constructed based on core-genome single nucleotide polymorphism sites by using 275 genomes, including 274 SL.

B Correlation of the phylogenetic tree with host, region and clades. Host of the isolates (Ring 1); geographical regions (Ring 2); the clade (majority ST) of the isolates (Ring 3). The black arrows indicate the isolates from feed imported to China.

Source data are available online for this figure.

 ©2022 The Authors

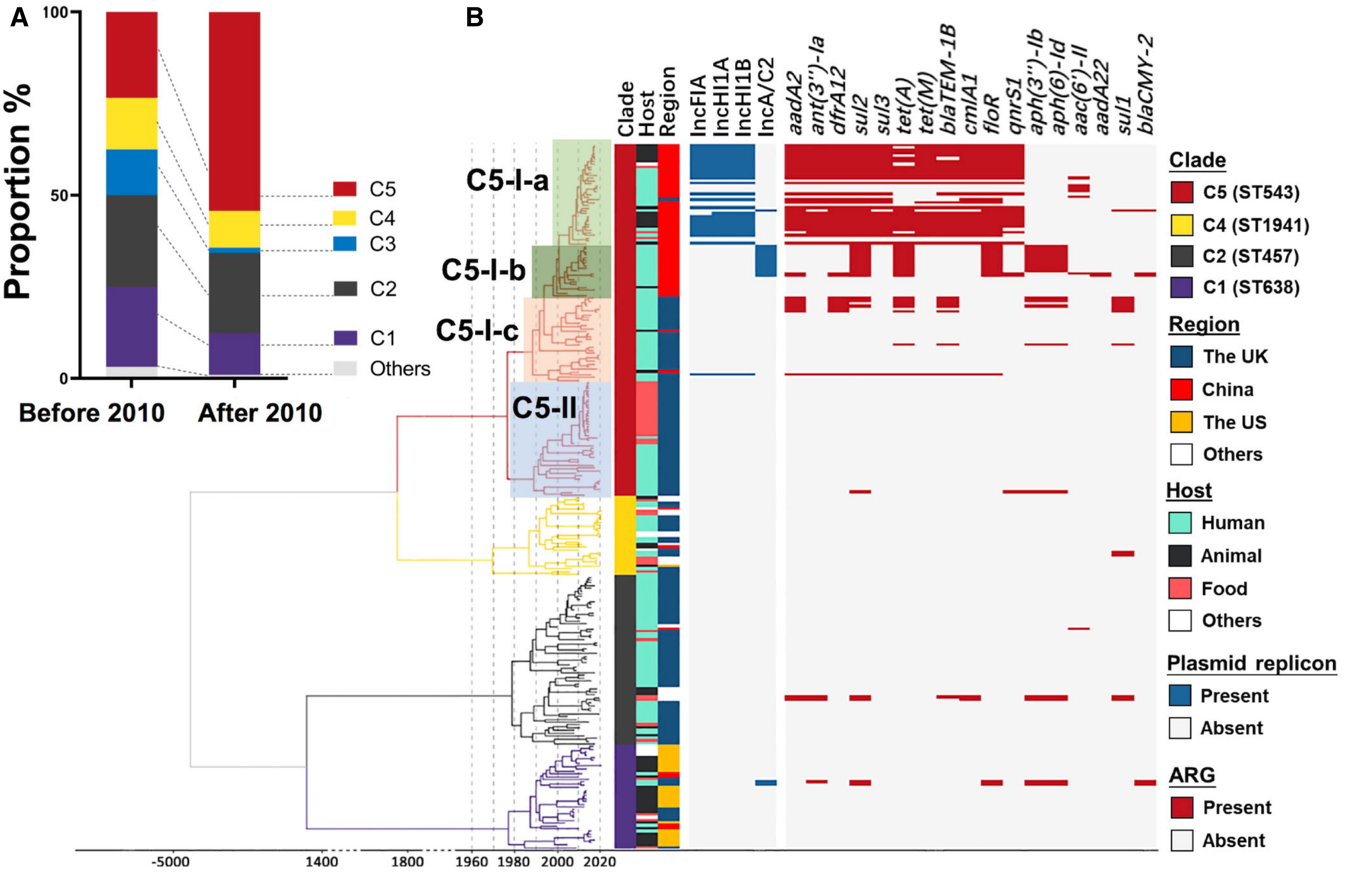

Figure 2. The recent emergence of multidrug-resistant SL in China.

A Proportion change of Chinese SL isolates from different phylogenetic clades. The Chinese isolates were classified into two groups based on their sampling time (1993–2010 vs. 2011–2020). The percentage of isolates from each clade in each period was compared.

B Bayesian inference of phylogeny. The time-calibrated Bayesian phylogenetic tree was built based on Core SNPs of SL strains by BEAST2 v2.6.3 (GTR substitution model, 4 gamma category count, relaxed clock log-normal model and Coalescent Bayesian Skyline tree prior model). The presence of plasmid replicon and ARGs were marked by blue and red blocks, respectively.

Source data are available online for this figure.

that animal feeds served as vehicles to introduce SL into China, likely for further dissemination via the food chain.

A recent expanding MDR clade in mainland China

We evaluated the MIC of 53 Chinese SL isolates against 16 antibiotics. More than half of the isolates were resistant or intermediate to ceftiofur (100%), tetracycline (86.8%), sulfamethoxazole-trimethoprim (71.7%), streptomycin (79.2%), chloramphenicol (77.4%) and ampicillin (58.5%) (Fig EV1; Table EV1). In total, 51 out of 53 isolates were MDR. All five iNTS strains (L39, L42, L43, L44 and 18B003) were MDR and phenotypically resistant to more than four classes of antibiotics.

To understand how the strain proportion of each clade changes, in particular, for AMR development, we compared the strain proportions of two time periods, before and after 2010. The ratios of C-1 to C-4 strains decreased after 2010, respectively, while the proportion of C-5 strains increased from 23.4% (15 cases) to 54.3% (114 cases) (Fig 2A), indicating a recent expansion from C-5.

To pinpoint the evolutionary history of the SL isolates, we assessed for temporal signals within these isolates from 1999 to 2020, followed by BEAST analysis. The sufficiency of the temporal signal was evaluated by a root-to-tip regression, resulting in removing the isolates from C-3 (n = 11) and C-4 (n = 4) with incongruent sampling date. Therefore, the remaining 258 strains were used to build the maximum clade credibility tree. Within C-5, strains were estimated to diverge into Clade-5-I (further as C-5-I-a, C-5-I-b, C-5-I-c) and Clade-5-II in ~1980 (Fig 2B; Dataset EV2). Most Chinese strains belong to C-5-I-a and C-5-I-b, which mainly comprised MDR isolates and emerged in ~2005 and ~2000, respectively. Notably, five iNTS isolates belong to the emerging C-5-I-a and C-5-I-b (Table EV1).

We further investigated and compared antimicrobial-resistant genes (ARGs). Notably, Chinese isolates harbour more ARGs than isolates of other countries, with 46.9% of Chinese isolates containing 10–13 ARGs (Fig EV2A). Increasing ARG numbers were observed in recent isolates (Fig EV3). The enriched ARGs in Chinese isolates confer resistance to a panel of antimicrobials, including

©2022 The Authors

aminoglycosides, sulphonamides, phenicols, tetracyclines, beta-lactams, quinolones and trimethoprim (Fig EV2B). Most of the isolates in C-1, C-2, C-3, C-4 and C-5-II contain no ARGs (average ARGs per isolate: C-1: 0.42, C-2: 0.35, C-3: 1.09, C-4: 0.13, C-5-II: 0.19), while the majority of the C-5-I-a strains, specifically Chinese strains, carried significantly more ARGs (average ARGs: 8.47; Dataset EV2). Interestingly, strains in three subclades of C-5-I showed distinct patterns of plasmid types and ARGs. The subclade C-5-I-c contained two strains from animal feed imported to China (L3 and L24) and showed the lowest number (average ARGs: 2). Besides, 57.9% of the strains in C-5-I-b that formed around the year 2000 carried an IncA/C2 plasmid and ARGs associated with resistance to sulphonamides, tetracyclines, quinolones and aminoglycosides (Dataset EV2). Surprisingly, 72.97% of the strains in C-5-I-a shared similar plasmid and ARG profiles, comprising a considerable number of plasmid types and ARGs. These included IncFIA, IncHI1A and IncHI1B replicons, and ARGs conferring resistance to aminoglycosides, sulphonamides, phenicols, tetracyclines, beta-lactams, quinolones and trimethoprim. The stepwise evolution of the Chinese strains from animal feed C-5-I-c strains to clinically C-5-I-b and C-5-I-a coincides with the acquisition of plasmids and ARGs.

The representative C-5-I-a (L35, L41 and lin-10) and C-5-I-b (L12 and L42) strains were confirmed to carry conjugative plasmids by conjugation assay, indicating an ARG transmission. Interestingly, the conjugation efficiencies were significantly different among strains from different subclades. Clade-5-I-b strains showed significantly higher efficiency rates (4.4×10^{-1} and 2.0×10^{-1} CFU per recipient cell for L12 and L42) than Clade-5-I-a strains (4.7×10^{-4}, 4.0×10^{-3} and 2.0×10^{-4} CFU per recipient cell for L35, L41 and lin-10, respectively, Appendix Table S1). The results indicate that horizontal gene transfer via conjugative plasmids played a role in the emergence of AMR subclades.

Clade-5 subgroups are regionally and phenotypically distinct

To provide comparative contextual analysis, we studied SL isolates from the French surveillance system in 1951, 1959 and 2016–2022. We found that the majority (55.0%) of French isolates belonged to C-5 ($n = 107$) and C-5-II ($n = 89$) in particular (Fig EV4A; Dataset EV3). From 2016 to 2021, C-5 and C-2 are leading clones circulating in French population, which were independently detected in China. To investigate the relatedness of C-5 isolates between France and China, we projected the phylogenetic tree by including all ST543 isolates (Fig EV4B). We detected that Chinese isolates mainly come from C-5-I-a and C-5-I-b, while French isolates mainly come from C-5-II and C-5-1-c. Additionally, the European isolates, including French and U.K. isolates, grouped into C-5-II and C-5-1-c, indicating strongly regional-associated clones among C-5 subgroups. Nevertheless, unique clones, C-5-I-a/b, were primarily found in China.

To understand whether the isolates were clade-specific in terms of the stress response, we investigated bacterial survival rates under oxidative and desiccation stresses. The growth rates of isolates from four clades were similar without significant difference (Appendix Fig S1); however, the C-5 (iNTS isolates L42 and L43) and C-4 isolates were significantly more resistant to hydrogen peroxide treatment than C-1 isolates (Fig 3A), suggesting improved survivability within macrophages. The C-1 isolates survived better than C-5 isolates under desiccation stresses (Fig 3B). The biofilm-forming capability of C-5 isolates is higher than that of C-1 and C-2 isolates but less than that of C-4 isolates (Fig 3C). Collectively, strains from different clades were phenotypically distinct.

In vivo niche adaptation for *Salmonella* Livingstone Clade-5-I-a/b

To understand whether C-5 invasive isolates are more virulent, we evaluated the survival rates of diverse animals, namely *Caenorhabditis elegans*, chicken embryos, zebrafish embryos and mice, after infecting them with isolates from two most phylogenetically distinct clades, C-5 and C-1. Although survival rates were similar in *C. elegans* (Appendix Fig S2), significant differences were observed in survival rates of chicken embryos and zebrafish (Fig 3D and E). C-5 iNTS isolates (e.g. L42 and L43) caused significantly higher deaths than C-1 isolates in chicken embryos 5 days postinfection (dpi). Similarly, compared with C-1 isolates, iNTS isolates of C-5 lead to significantly higher number of zebrafish deaths (Fig 3E). Notably, none of the isolates from the two clades killed mice after an oral challenge, whereas a dramatic difference in bacterial loads within their organs was detected. The bacterial load in the liver and cecum of infected mice was significantly higher for C-5 than that of C-1, both on day three and five postinfection, while no significant difference was observed in the spleen (Fig 3F). Together, we found that the emerging Chinese C-5-I-a/b might be more virulent for vertebrates.

To understand the genetic factors contributing to the virulence of C-5 isolates, we defined and compared the virulent genes (VGs) of isolates from each clade (Dataset EV4). Differences in VG profiles were observed between isolates from C-5 to C-1. Compared with the C-1 isolates, C-5 isolates, including iNTS isolates, contained *sopD2* (a type III secretion system effector) and *ratB* (a colonisation factor) but lacked the *lpf* operon (bacterial fimbriae).

Discussion

Bloodstream infections caused by iNTS result in enormous public health concerns worldwide, as they are frequently associated with poor outcomes (Paudyal *et al*, 2020; Shi *et al*, 2021). Salmonellosis caused by iNTS has been increasingly reported in China, though the mechanisms were rarely investigated (Yue *et al*, 2021). Here, SL was used as a model to understand the dynamics of iNTS in China. Our data demonstrate that the emergence of iNTS caused by SL in clinical cases was somewhat unexpected, as few studies since 1953 provided evidence for such a phenomenon (Picton & Stirrup, 1953). Notably, the international trade of animal feed contaminated with SL was identified as a possible import vehicle into China. Remarkably, during its short adaption in China, we observed the emerging MDR and hypervirulent subclades of SL with an acquired ARG-carrying plasmid and niche-specific adaptation evidenced by increased pathogenicity towards chicken, zebrafish and mice.

Based on genomic investigation of SL from various sources, we propose that animal feed could be the vehicle imported to China. Although only five isolates originated from imported fish meals in our study, they were distributed over three distinct clades (C1, C4 and C5 in Fig 1B), indicating the fish meals could serve as a reservoir of versatile genetically distinct SL. Notably, the detection of SL in fish meals imported to China was reported in the 1990s (Chen

 ©2022 The Authors

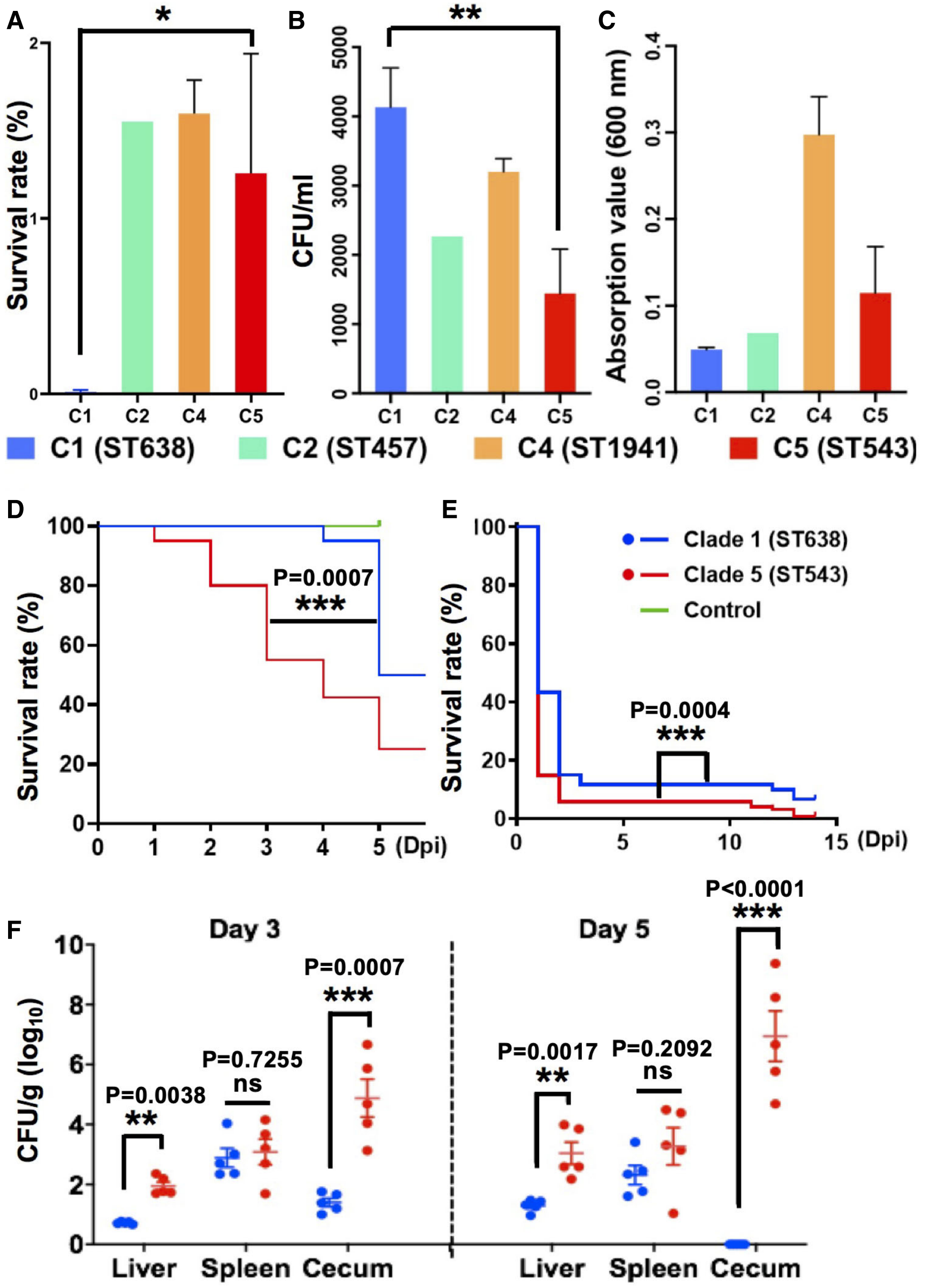

Figure 3. Niche-specific adaptation for SL Clade 5 isolates.

A Bacterial resistance to desiccation. Representative isolates that are phylogenetically distinct within Clade-1 (n = 2), Clade-2 (n = 1), Clade-4 (n = 2) and Clade-5 (n = 6) were used for desiccation assay, as well as following hydrogen peroxide assay (B) and biofilm assay (C).

B Bacterial survivability under oxidative stress. Representative isolates from C1 (n = 2), C2 (n = 1), C4 (n = 2) and C5 (n = 6) were treated with hydrogen peroxide.

C Biofilm formation ability. The biofilm of the representative isolates from C1 (n = 2), C2 (n = 1), C4 (n = 2) and C5 (n = 6) were formed at 22°C for 48 h.

D Comparison of survival rates of chick embryo after challenging SL C5 and C1 isolates. Comparison was on 6 days post-infection (dpi). Ten embryos were used for each group.

E Survival rates of zebrafish embryo after challenging SL C5 and C1 isolates. The comparison was on 14 dpi. Ten zebrafish embryos were used for each group.

F Bacterial loads in distinct organs of mice after challenging SL C5 (L42) and C1 (L1) isolates. The bacterial counts in the liver, spleen and cecum of mice were measured 3 and 5 dpi. Ten mice were used per group.

Data information: All the experiments were triplicated with biological replicates. Data are presented as mean ± SD. The P-values for (A–C) and (F) were calculated using an unpaired t-test (two-tailed), and P-values for (D) and (E) were calculated using the log-rank (Mantel–Cox, chi-square) test (*P < 0.05, **P < 0.01, ***P < 0.001). Source data are available online for this figure.

et al, 1994), and the amount of imported fish meals increased 90 times during the past four decades (IndexMundi, 2022), highlighting that the fish meals is a transfer vehicle for SL spreading. In support of international feed-borne transmission, a recent study demonstrated the strong survivability of SL in a low-moisture environment (Abdelhamid & Yousef, 2021), in consistence with high resistance to desiccation (fish meal environments) for our fish meals-derived C-1 isolates (isolate L1 from fish meal imported to China, Fig 3). Except for the imported animal feed, the swine supply chain is a plausibly additional niche for MDR development, supported by MDR isolates from swine carcasses and the environment of slaughterhouses, as shown by previous studies (Bonardi *et al*, 2016; Colello *et al*, 2018). The largest swine supply chain in China amplifies foodborne transmission, while fish meals are frequently supplemented as feed in the modern swine industry (Kim & Easter, 2001). Notably, aquatic animals were also found to carry this pathogen (Dataset EV2; Guerin *et al*, 2004). Extensive evidence documented this pathogen in seafood, fish, fish meals or feedstuffs from Northern Europe (Katouli *et al*, 1994; Nesse *et al*, 2005), Western Asia (Nabbut *et al*, 1982) and Southeast Asia (Heinitz *et al*, 2000).

Phenotypic and genotypic analyses indicate emerging C-5 isolates from China have evolved in a manner of enhancing pathogenicity. Notably, the C-5 isolates displayed significantly elevated virulence in chicken embryos and mice compared with C-1 (Fig 3C–E). This is in concordance with the presence of C-5 specific VGs, including *sopD2* and *ratB* (Dataset EV4), suggesting they may contribute to the invasive feature of C-5 isolates. Interestingly, deletion of *sopD2* in *S*. Typhimurium showed decreasing bacterial replication within mouse macrophages and the spleen of infected mice (Jiang *et al*, 2004). The presence of *sopD2* in SL C-5 isolates might explain the higher bacterial load in the spleen of mice compared with C-1 (Fig 3F). Besides, Kingsley *et al* (2003), demonstrated the *ratB* deletion mutant of *S*. Typhimurium showed a defect for colonisation of the mice cecum. Further study is needed for understanding clade- or subclade-specific virulence.

Acquisition of AMR is a significant feature for C-5-I-b and C-5-I-a. These two subclades related to MDR only consisted of Chinese isolates and emerged in the 1990s and 2000s, respectively (Fig 2B). The isolates at the root of C-5-I-b without ARG or plasmids evolved into MDR by obtaining ARGs through plasmid acquisition. Although we cannot rule out the possibility that the use of antibiotics in humans caused the selection of two subclades, the shift of Chinese isolates to MDR is plausibly driven by the extensive use of antibiotics in animal farming. Indeed, 81.1% of Chinese isolates resist ceftiofur, a 3rd-generation cephalosporin used explicitly for farm animals (Lai *et al*, 2014). This is consistent with the fact that most Chinese isolates carry β-lactamase encoding genes (bla_{CMY-2}) (Fig 2B) and conjugative plasmids, highlighting that the antibiotic-containing feed and overuse of antibiotics could promote the selection of AMR. An astonishing finding is that SL isolates from China were resistant to more antibiotics and carried a higher number of ARGs than isolates from other countries. These findings emphasise the need for urgent actions to control AMR in China (Yue *et al*, 2020; Wang *et al*, 2022).

Collectively, we observed a significant within-serovar variability of SL population concerning MDR and invasive potentials, which was tightly associated with specific clades. The detection of emerging invasive isolates from C-5-I-a/b highlights the power of One Health genomic surveillance.

Materials and Methods

Ethics statements

The research protocol for mice, chicken and zebrafish infection experiments conducted in this study was approved by the Committee of Laboratory Animal Center of Zhejiang University (ZJU20190094 and ZJU20170360).

The examined *Salmonella* isolates were obtained from a local surveillance programme by the Shanghai Municipal CDC and Zhejiang CDC (with ethical approval 2019-014), following the recommendations of the Chinese National CDC. The invasive cases were collected as part of a national-wide invasive *Salmonella* infection project, which recruited bloodstream infections in over 20 provinces or municipal cities in China. Informed written consent for using surveillance samples was obtained from the patients or guardians.

To provide a comparative cohort with the global contextual collection, 196 human isolates and one blood-meal isolate were collected by the Unité des Bactéries Pathogènes Entériques, Institut Pasteur, Paris, France. The remaining strains and their corresponding metadata, if available, are published online with ethical approval.

Antimicrobial susceptibility testing

To analyse the antimicrobial drug susceptibility of 53 isolates from China, we examined 16 antimicrobial agents, including ampicillin (AMP), amoxicillin-clavulanic acid (AMC), ceftriaxone (CRO), ceftiofur (TIO), cefoxitin (FOX), imipenem (IPM), gentamicin (GEN), kanamycin (KAN), streptomycin (STR), tetracycline (TET), ciprofloxacin (CIP), nalidixic acid (NAL), trimethoprim-sulfamethoxazole (SXT), colistin (CST), azithromycin (AZM) and chloramphenicol (CHL), under aerobic condition (Wu *et al*, 2021). Minimal inhibitory concentrations (MIC) were determined by the broth microdilution assay, according to the criteria recommended by the Clinical and Laboratory Standards Institute (CLSI) and National Antimicrobial Resistance Monitoring System for Enteric Bacteria (NARMS; https://www.cdc.gov/narms/antibiotics-tested.html; CLSI, 2016). Except for ceftiofur and streptomycin, the breakpoints for the antibiotics were from CLSI. The breakpoints for ceftiofur and streptomycin were from NCCLS and NARMS, respectively. *Escherichia coli* ATCC 25922 was used as a control strain.

Bacterial strains and whole genome sequencing

The primary dataset contains 274 SL genomes, including 221 publicly available genome sequences from Enterobase (www.enterobase.warwick.ac.uk; Zhou *et al*, 2020) and 53 isolates from China, which were obtained in the course of the current study. These 53 SL isolates were collected in mainland China between 1999 and 2018 from various locations, including hospitals, slaughterhouses, farms and markets (Dataset EV1).

The cohort SL isolates (n = 211), with complete metadata, were provided by Institute Pasteur, France (Dataset EV3). One hundred ninety-six of these human isolates were recruited as part of routine surveillance by the French-national *Salmonella* reference laboratory between Jan 2016 and Jan 2022.

The serum agglutination tests were performed on all examined isolates according to the Kaufmann-White classification to confirm

©2022 The Authors

the Livingstone serovar, as described previously (Liu *et al*, 2021; Wu *et al*, 2021). The genomic DNA was extracted using a commercial genomic DNA extraction kit according to the manufacturer's protocol. Whole genome sequencing (WGS) was performed by using Illumina NovaSeq 6000 to generate 150 bp paired-end reads. The short read datasets were submitted to the NCBI Sequencing Read Archive under BioProject PRJNA813213.

Genome assemblies and genetic screening

The raw reads were checked for sequencing quality by FastQC and trimmed using Trimmomatic (Babraham-Bioinformatics, 2011; Bolger *et al*, 2014). *De novo* assembly was performed to generate contigs using SPAdes (Bankevich *et al*, 2012). *In silico* serotyping of all genomic sequences was performed by SeqSero2 (Zhang *et al*, 2019). The genomic sequences of 274 SL isolates were screened for multilocus sequence types (MLSTs) using seven housekeeping genes by MLST ver. 2.16.1. Antimicrobial-resistant genes (ARGs), plasmids and virulence genes were identified using CGE ResFinder (ver. 3.1) (DNA identity ≥ 90%, coverage ≥ 60%) (Bortolaia *et al*, 2020), CGE PlasmidFinder (ver. 2.0) (DNA identity ≥ 95%, coverage ≥ 60%) (Carattoli & Hasman, 2020) and VFDB (DNA identity ≥ 90%, coverage ≥ 60%) embedded in Abricate, respectively, as previously described with minor modification (Teng *et al*, 2019, 2020). To predict the location of AMR genes, i.e., plasmid or chromosome, we assembled the plasmid contigs from the sequencing reads using plasmidSPAdes and screened for ARGs on assembled plasmid sequences by Resistance Gene Identifier (RGI; Peng *et al*, 2022).

Phylogenetic analysis

The phylogeny of 274 global SL isolates was conducted as previously described (Li *et al*, 2022). Briefly, the genomic sequences were mapped to the closed genome of SL SA20101045 using Snippy v4.4.4, followed by extracting core-genome single nucleotide polymorphisms (SNPs). The SNPs from the recombination regions were detected by Gubbins v2.3.4 and filtered using Snippy v4.4.4. A maximum-likelihood phylogenetic tree was constructed based on the filtered core-genome SNPs using the best model (TVM+F) detected by IQ-TREE. The phylogenetic tree was rooted in an outgroup strain, SAL00036 (SRR12528070). The French cohort isolates (n = 196), as well as the representative isolates from lineages, were used the same parameters to project the phylogeny tree.

To investigate the temporal signal of SL genomes, we used TempEst v1.5.3 (Rambaut *et al*, 2016). The phylogenetic tree was constructed based on BEAST2 platform (Bouckaert *et al*, 2014), using the GTR substitution model, 4 Gamma Category Count, Relaxed Clock Log-Normal model and Coalescent Bayesian Skyline tree prior model was used for the time-scaled tree. A Markov Chain Monte Carlo chain length of 100,000,000 was configured. The phylogenetic tree was projected using the iTOL platform and R v4.0.5.

Conjugation assay

A conjugation assay was conducted according to our previous study (Elbediwi *et al*, 2020; Liu *et al*, 2021). Representative clinical isolates (L12, L35, L41, L42 and Lin-10) served as donor strains, and *E. coli* EC600 with rifampicin resistance was used as a recipient strain. The donor and recipient isolates were cultured overnight at 37°C, followed by dilution in LB to approximately 10^8 CFU/ml. The donor (1 ml) and recipient (1 ml) isolates were mixed with 4 ml of LB broth and incubated at 37°C for 24 h, followed by serial 10-fold dilutions using PBS. Xylose Lysine Deoxycholate agar plates containing rifampicin (50 μg/ml) were used to select recipients, and Xylose Lysine Deoxycholate agar plates supplemented with rifampicin (50 μg/ml) and tetracycline (16 μg/ml) were used to select successful transconjugants. Conjugation frequency was calculated by dividing the number of transconjugants by the number of recipients.

Phenotypic comparison of growth rate, biofilm formation and resistance to desiccation and hydrogen peroxide

To further explore the phenotypic diversity of strains from different clades, a series of representative isolates (Clade-1: strain L1 and L6; Clade-2: strain L45; Clade-3: strain L9 and L23; Clade-5: Clade-5-I-a strain L22 and L32, Clade-5-I-b strain L42 and L43, Clade-5-I-c strain L3 and L24) were selected to test their growth rate, biofilm formation and resistance to hydrogen peroxide and desiccation. To determine the growth rate of these strains, bacteria were cultured overnight in LB broth. Then, the bacterial solutions were diluted 1:100 in 15 ml LB broth and incubated at 37°C. The absorbance of the bacterial culture was measured each hour with three biological replicates.

The desiccation assay was conducted following the previous procedure with minor modification (Li *et al*, 2022). The overnight cultures (LB, 37°C, shaking for 18–24 h) of the isolates were seeded to LB media at a ratio of 1:100, and the subcultures were incubated with shaking at 37°C until the OD_{600} reached one. Afterwards, the bacterial cultures were washed and suspended with 10 mM PBS, and 50 μl of suspensions were added to 96-well polystyrene plates. The plates were incubated at 22°C for 24 h in a sealed container with saturated potassium acetate solution to maintain a relative humidity of 36%. To count bacterial concentration, the initial and final bacterial cultures were serially 10-fold diluted and spread on LB agar plates, followed by enumeration. The survival rate under desiccation was calculated by dividing the final bacterial count by the initial bacterial count. Statistical analysis was conducted using an unpaired t-test.

The biofilm assay was conducted as previously described (Li *et al*, 2022). The overnight culture (LB, 37°C, shaking for 18–24 h) of the representative isolates was adjusted to OD_{600} = 0.4, and the adjusted bacterial culture was the seed to LB at a ratio of 1:100 to make a subculture. After adding 200 μl of subculture to 96-well polystyrene microtiter plates (five parallel wells for each isolate), the plates were still placed at 22°C for 48 h. Then, the bacterial cultures were washed three times using ddH_2O, followed by drying the plates and adding 200 μl of 0.4% crystal violet to each well for 25 min. Finally, a mixture of ethanol and acetone (3:1) was applied for destaining for 20 min, and the absorbance values of the destaining solutions were measured using a microplate reader at OD_{590}. *Escherichia coli* ATCC 27853 was used as a control strain in the biofilm formation test. Statistical analysis was conducted using an unpaired t-test.

The hydrogen peroxide resistance was evaluated as previously described with minor modifications (Hayden *et al*, 2016). The initial absorbance of the bacterial solution was adjusted to 0.4–0.5 (OD_{600}) at the stationary phase. Then, the cell suspension was incubated for 40 min in phosphate-buffered saline pH 7.4 with 4 mM hydrogen

©2022 The Authors

peroxide, at 37°C. The control group was set without hydrogen peroxide. Serial 10-fold dilutions were cultured on LB agar to determine viable cell counts. The percentage survival of each strain was calculated using the following formula: (average number of colonies in the experimental group/average number of colonies in the control group) × 100%. *P*-values were calculated by unpaired *t*-test.

Pathogenicity assays in four animal models

To investigate the pathogenicity of SL from different clades, four animal models, including *C. elegans*, chicken, zebrafish and mice, were used with the bacteria as previously described with minor modifications. The *C. elegans*, strain SS104 genotype *glp-4* (bn2), was maintained based on the previous description (Stiernagle, 1999; Xu *et al*, 2020). When the *C. elegans* were cultured to the larval-2 stage, all worms were transferred to the NGM plates with distinct bacterial lawns (Clade-1 strain L1 and L6, Clade-5-I-a strain L22 and L32; and Clade-5-I-b strain L42 and L43) and *E. coli* OP50 served as a control. The number of dead worms was checked every day and the living worms were transferred to new plates. A total of 210 worms (three biological replicates of 10 embryos) were infected.

The chicken embryo model was also used to test bacterial pathogenicity based on a previous protocol with modification (Li *et al*, 2019). A total of 70 SPF chicken embryos were incubated for 16 days at 37°C and 60–70% humidity. Six isolates from Clade-1 (L1 and L6) and Clade-5 (Clade-5-I-a strain L22 and L32; Clade-5-I-b strain L42 and L43) were cultured in the early stationary phase. The bacterial culture (1×10^2 CFU) of each isolate was injected into 10 embryos. The embryos injected with PBS were used as a positive control. The growth of embryos was observed every day for 5 days until the birth of the chicken. The experiment was repeated three times, and the survival curves were analysed by the log-rank (Mantel–Cox, chi-square) test.

The zebrafish embryo microinjection model was set up for the virulence assay, and zebrafish (*Danio rerio*, genotype AB) were used in this study (Tan *et al*, 2019). AB zebrafish larvae at 3 dpi were anaesthetized in E3 medium, placed on an agarose plate and individually microinjected using pulled glass microcapillary pipettes. Larvae were microinjected with an average of 10 CFU via yolk sac, following the recommendations described (Benard *et al*, 2012; Shan *et al*, 2015). The control group was injected with PBS. The examined groups were injected with the same volume from Clade-1 (L1 and L6) and Clade-5 (L22, L32, L42 and L43). The infected and control larvae were maintained at 28°C in 6 ml of E3 egg water. Animals were regularly observed, and cumulative mortalities were registered until 14 dpi. A total of 210 animals (three biological replicates of 10 embryos) were infected. Statistical analysis was conducted by Log-rank (Mantel–Cox, chi-square) test.

Besides, we evaluated the survival rate of mice after infection and measured the bacterial loads in their tissue (Wu *et al*, 2010; Nilsson *et al*, 2019). The C57BL/6 female mice, purchased from Beijing Vital River Laboratory Animal Technology Co., Ltd., were separated into three groups with 10 mice in each group at 26°C. The mice were orally challenged with a volume of 1×10^8 CFU bacteria (Clade-1 strain L1 and Clade-5-I-b strain L42). The survival of the mice was observed every day for 5 days. On day 3 and 5, three mice from each group were randomly selected to collect their livers, spleens and cecum. These organs were homogenated with sterile tetragonal zirconium polycrystal, followed by 10-fold serial dilutions and plating on LB agar. The agar plates were incubated at 37°C overnight, and colonies were enumerated to calculate bacterial loads in different tissue. The experiments were triplicated, and statistical analyses for bacterial counts were conducted using an unpaired *t*-test.

The paper explained

Problem

Invasive nontyphoidal *Salmonella* (iNTS), including *Salmonella* enterica serovar Livingstone (*S.* Livingstone), is an important group of isolates that cause life-threatening invasive infections in humans (e.g. bloodstream infection). In recent years, these cases were increasingly observed in clinical settings in China, and *S.* Livingstone has been emerging as a causative agent, suggesting their enhanced adaptation to humans. However, the source and evolutionary trajectory of these pathogens remain unclear.

Results

This study identified imported animal feed, as well as livestock and aquatic animals, as a major source of *S.* Livingstone in China, indicating international animal feed trade is a vehicle to introduce this pathogen into China. In a global context, the Chinese clinical isolates mainly belong to the Clade-5 (C-5), which has been expanding in China along with enhanced resistance to desiccation and diminished tolerance to oxidative stress, compared with the ancestor Clade-1 isolates. Of note, our study for the first time reported that two novel C-5 subgroups (i.e. C-5-I-a/b) associated with human infections substantially emerged in mainland China in the last ~20 years, accompanying accumulated antimicrobial resistance. These newly occurred isolates showed augmented virulence in animal infection models (i.e. chicken embryos, zebrafish embryos and mice), indicating their evolution in a path towards enhanced host adaptation.

Impact

Our findings provide genotypic and phenotypic evidence to understand when and how the novel human infection-related *S.* Livingstone clades emerge in China. Given that the isolates of these novel clades showed enhanced virulence and were associated with antimicrobial treatment failure, routine surveillance for these pathogens in animal husbandries, food processing chains and healthcare settings is highly needed.

Statistical analysis

GraphPad Prism 9.0 software was used for statistical analyses of *in vivo and in vitro* experiments (i.e., "Phenotypic comparison of growth rate, biofilm formation and resistance to desiccation and hydrogen peroxide" and "Pathogenicity assays in four animal models"). Details of statistical tests performed for each experiment are mentioned in the "Materials and Methods" and "Figure legends" sections.

Data availability

All the sequencing data of the SL isolates from this study are deposited in Sequence Read Achieve of NCBI under BioProject number PRJNA813213 at https://www.ncbi.nlm.nih.gov/sra/?term=PRJNA 813213 (Dataset EV1).

Expanded View for this article is available online.

©2022 The Authors

Acknowledgements

This study was supported by the National Program on Key Research Project of China (2019YFE0103900 and 2017YFC1600103) as well as the European Union's Horizon 2020 Research and Innovation Programme under Grant Agreement No. 861917—SAFFI, National Natural Science Foundation of China (31872837, 32150410374), Zhejiang Provincial Natural Science Foundation of China (LR19C180001), Zhejiang Provincial Key R&D Program of China (2022C02024, 2021C02008) and Hainan Provincial Joint Project of Sanya Yazhou Bay Science and Technology City (2021JJLH0083).

Author contributions

Yan Li: Conceptualization; resources; data curation; supervision; funding acquisition; investigation; methodology; writing – review and editing. **Lin Teng:** Conceptualization; data curation; formal analysis; validation; investigation; visualization; methodology; writing – original draft; writing – review and editing. **Xuebin Xu:** Conceptualization; resources; data curation; writing – review and editing. **Xiaomeng Li:** Data curation; validation; investigation; visualization; methodology; writing – original draft. **Xianqi Peng:** Investigation; methodology. **Xiao Zhou:** Investigation; methodology. **Jiaxin Du:** Investigation; methodology. **Yanting Tang:** Investigation; methodology. **Zhijie Jiang:** Investigation; methodology. **Zining Wang:** Investigation; methodology. **Chenghao Jia:** Investigation; methodology. **Anja Müller:** Investigation; writing – review and editing. **Corinna Kehrenberg:** Investigation; writing – review and editing. **Haoqiu Wang:** Resources; data curation. **Beibei Wu:** Resources; data curation. **François-Xavier Weill:** Resources; investigation; writing – review and editing. **Min Yue:** Conceptualization; resources; data curation; supervision; funding acquisition; validation; investigation; project administration; writing – review and editing.

Disclosure and competing interests statement

The authors declare that they have no conflict of interest.

For more information

a. https://pubmed.ncbi.nlm.nih.gov/35717143/
b. https://wwwnc.cdc.gov/travel/yellowbook/2020/travel-related-infectious-diseases/salmonellosis-nontyphoidal
c. https://www.who.int/news-room/fact-sheets/detail/salmonella-(non-typhoidal)
d. https://person.zju.edu.cn/en/myue

References

Abdelhamid AG, Yousef AE (2021) Natural antimicrobials suitable for combating desiccation-resistant *Salmonella enterica* in milk powder. *Microorganisms* 9: 421

Bankevich A, Nurk S, Antipov D, Gurevich AA, Dvorkin M, Kulikov AS, Lesin VM, Nikolenko SI, Pham S, Prjibelski AD *et al* (2012) SPAdes: a new genome assembly algorithm and its applications to single-cell sequencing. *J Comput Biol* 19: 455–477

Benard EL, van der Sar AM, Ellett F, Lieschke GJ, Spaink HP, Meijer AH (2012) Infection of zebrafish embryos with intracellular bacterial pathogens. *J Vis Exp* 61: 3781

Babraham-Bioinformatics (2011) *FastQC: a quality control tool for high throughput sequence data*. Cambridge: Babraham Institute

Bolger AM, Lohse M, Usadel B (2014) Trimmomatic: a flexible trimmer for illumina sequence data. *Bioinformatics* 30: 2114–2120

Bonardi S, Bruini I, Alpigiani I, Vismarra A, Barilli E, Brindani F, Morganti M, Bellotti P, Bolzoni L, Pongolini S (2016) Influence of pigskin on *Salmonella* contamination of pig carcasses and cutting lines in an Italian slaughterhouse. *Ital J Food Saf* 5: 5654

Bortolaia V, Kaas RS, Ruppe E, Roberts MC, Schwarz S, Cattoir V, Philippon A, Allesoe RL, Rebelo AR, Florensa AF *et al* (2020) ResFinder 4.0 for predictions of phenotypes from genotypes. *J Antimicrob Chemother* 75: 3491–3500

Bouallègue-Godet O, Salem Youssef B, Fabre L, Demartin M, Grimont Patrick AD, Mzoughi R, Weill F-X (2005) Nosocomial outbreak caused by *Salmonella enterica* serotype Livingstone producing CTX-M-27 extended-Spectrum β-lactamase in a neonatal unit in Sousse, Tunisia. *J Clin Microbiol* 43: 1037–1044

Bouckaert R, Heled J, Kühnert D, Vaughan T, Wu C-H, Xie D, Suchard MA, Rambaut A, Drummond AJ (2014) BEAST 2: a software platform for Bayesian evolutionary analysis. *PLoS Comput Biol* 10: e1003537

Carattoli A, Hasman H (2020) PlasmidFinder and *in silico* pMLST: identification and typing of plasmid replicons in whole-genome sequencing (WGS). *Methods Mol Biol* 2075: 285–294

Chen B, Huang J, Chen B, Shen X, Chen Z, Zheng G (1994) Report of *Salmonella* in imported animal products. *Entry & Exit Animal & Plant Quarantine of China* 3: 28–30

CLSI (2016) *Performance standards for antimicrobial susceptibility testing CLSI supplement M100S*, 26th edn. Wayne, PA: Clinical and Laboratory Standards Institute

Colello R, Ruiz MJ, Padin VM, Roge AD, Leotta G, Padola NL, Etcheverria AI (2018) Detection and characterization of *Salmonella* serotypes in the production chain of two pig farms in Buenos Aires Province, Argentina. *Front Microbiol* 9: 1370

GBD 2017 Non-Typhoidal Salmonella Invasive Disease Collaborators (2019) The global burden of non-typhoidal *Salmonella* invasive disease: a systematic analysis for the global burden of disease study 2017. *Lancet Infect Dis* 19: 1312–1324

Elbediwi M, Beibei W, Pan H, Jiang Z, Biswas S, Li Y, Yue M (2020) Genomic characterization of *mcr-1*-carrying *Salmonella enterica* serovar 4,[5],12:i:- ST 34 clone isolated from pigs in China. *Front Bioeng Biotechnol* 8: 663

Guerin PJ, De Jong B, Heir E, Hasseltvedt V, Kapperud G, Styrmo K, Gondrosen B, Lassen J, Andersson Y, Aavitsland P (2004) Outbreak of *Salmonella* Livingstone infection in Norway and Sweden due to contaminated processed fish products. *Epidemiol Infect* 132: 889–895

Hayden HS, Matamouros S, Hager KR, Brittnacher MJ, Rohmer L, Radey MC, Weiss EJ, Kim KB, Jacobs MA, Sims-Day EH *et al* (2016) Genomic analysis of *Salmonella enterica* serovar Typhimurium characterizes strain diversity for recent U.S. salmonellosis cases and identifies mutations linked to loss of fitness under nitrosative and oxidative stress. *mBio* 7: e00154

Heinitz ML, Ruble RD, Wagner DE, Tatini SR (2000) Incidence of *Salmonella* in fish and seafood. *J Food Prot* 63: 579–592

Hu B, Hou P, Teng L, Miao S, Zhao L, Ji S, Li T, Kehrenberg C, Kang D, Yue M (2022) Genomic investigation reveals a community typhoid outbreak caused by contaminated drinking water in China, 2016. *Front Med (Lausanne)* 9: 753085

IndexMundi (2022) China fish meal imports by year. https://www.indexmundi.com/agriculture/?country=cn&commodity=fish-meal&graph=imports (accessed on 15 August 2022)

Jiang X, Rossanese OW, Brown NF, Kujat-Choy S, Galan JE, Finlay BB, Brumell JH (2004) The related effector proteins SopD and SopD2 from *Salmonella enterica* serovar Typhimurium contribute to virulence during systemic infection of mice. *Mol Microbiol* 54: 1186–1198

©2022 The Authors

Johnson R, Mylona E, Frankel G (2018) Typhoidal *Salmonella*: distinctive virulence factors and pathogenesis. *Cell Microbiol* 20: e12939

Katouli M, Wollin R, Gunnarsson A, Kuhn I, Mollby R (1994) Biochemical phenotypes of *Salmonella* Livingstone isolated from humans, animals and feedstuffs in Sweden. *Acta Vet Scand* 35: 27 – 36

Kim SW, Easter RA (2001) Nutritional value of fish meals in the diet for young pigs. *J Anim Sci* 79: 1829 – 1839

Kingsley RA, Humphries AD, Weening EH, De Zoete MR, Winter S, Papaconstantinopoulou A, Dougan G, Baumler AJ (2003) Molecular and phenotypic analysis of the CS54 Island of *Salmonella enterica* serotype Typhimurium: identification of intestinal colonization and persistence determinants. *Infect Immun* 71: 629 – 640

Klemm EJ, Gkrania-Klotsas E, Hadfield J, Forbester JL, Harris SR, Hale C, Heath JN, Wileman T, Clare S, Kane L *et al* (2016) Emergence of host-adapted *Salmonella* enteritidis through rapid evolution in an immunocompromised host. *Nat Microbiol* 1: 1 – 6

Lai J, Wu C, Wu C, Qi J, Wang Y, Wang H, Liu Y, Shen J (2014) Serotype distribution and antibiotic resistance of *Salmonella* in food-producing animals in Shandong province of China, 2009 and 2012. *Int J Food Microbiol* 180: 30 – 38

Li Q, Li Y, Xia J, Wang X, Yin K, Hu Y, Yin C, Liu Z, Jiao X (2019) Virulence of *Salmonella enterica* serovar Pullorum isolates compared using cell-based and chicken embryo infection models. *Poult Sci* 98: 1488 – 1493

Li Y, Ed-Dra A, Tang B, Kang X, Muller A, Kehrenberg C, Jia C, Pan H, Yang H, Yue M (2022) Higher tolerance of predominant *Salmonella* serovars circulating in the antibiotic-free feed farms to environmental stresses. *J Hazard Mater* 438: 129476

Liu Y, Jiang J, Ed-Dra A, Li X, Peng X, Xia L, Guo Q, Yao G, Yue M (2021) Prevalence and genomic investigation of *Salmonella* isolates recovered from animal food-chain in Xinjiang, China. *Food Res Int* 142: 110198

Marchello CS, Birkhold M, Crump JA, Vacc-iNTS Consortium Collaborators (2022) Complications and mortality of non-typhoidal *Salmonella* invasive disease: a global systematic review and meta-analysis. *Lancet Infect Dis* 22: 692 – 705

Nabbut NH, Barbour EK, Al-Nakhli HM (1982) *Salmonella* species and serotypes isolated from farm animals, animal feed, sewage, and sludge in Saudi Arabia. *Bull World Health Organ* 60: 803 – 807

Nesse LL, Refsum T, Heir E, Nordby K, Vardund T, Holstad G (2005) Molecular epidemiology of *Salmonella* spp. isolates from gulls, fish-meal factories, feed factories, animals and humans in Norway based on pulsed-field gel electrophoresis. *Epidemiol Infect* 133: 53 – 58

Nilsson OR, Kari L, Steele-Mortimer O (2019) Foodborne infection of mice with *Salmonella* Typhimurium. *PLoS One* 14: e0215190

Old D, Porter-Boveri M, Munro D (1994) Human infection in Tayside, Scotland due to *Salmonella* serotype Livingstone. *J Med Microbiol* 40: 139 – 140

Paudyal N, Pan H, Wu B, Zhou X, Zhou X, Chai W, Wu Q, Li S, Li F, Gu G *et al* (2020) Persistent asymptomatic human infections by *Salmonella enterica* serovar newport in China. *mSphere* 5: e00163-20

Peng X, Ed-Dra A, Yue M (2022) Whole genome sequencing for the risk assessment of probiotic lactic acid bacteria. *Crit Rev Food Sci Nutr*: 1 – 19

Picton WH, Stirrup W (1953) A new *Salmonella* type, *Salm*. Livingstone. *J Pathol Bacteriol* 66: 310 – 312

Qiu YF, Nambiar RB, Xu XB, Weng ST, Pan H, Zheng KC, Yue M (2021) Global genomic characterization of *Salmonella enterica* serovar Telelkebir. *Front Microbiol* 12: 704152

Rambaut A, Lam TT, Max Carvalho L, Pybus OG (2016) Exploring the temporal structure of heterochronous sequences using TempEst (formerly Path-O-Gen). *Virus Evol* 2: vew007

Shan Y, Fang C, Cheng C, Wang Y, Peng J, Fang W (2015) Immersion infection of germ-free zebrafish with *Listeria monocytogenes* induces transient expression of innate immune response genes. *Front Microbiol* 6: 373

Shi D, Anwar TM, Pan H, Chai W, Xu S, Yue M (2021) Genomic determinants of pathogenicity and antimicrobial resistance for 60 global *Listeria monocytogenes* isolates responsible for invasive infections. *Front Cell Infect Microbiol* 11: 718840

Stiernagle T (1999) Maintenance of *C. elegans*. *WormBook* 2: 51 – 67

Tan F, Limbu SM, Qian Y, Qiao F, Du ZY, Zhang M (2019) The responses of germ-free zebrafish (*Danio rerio*) to varying bacterial concentrations, colonization time points, and exposure duration. *Front Microbiol* 10: 2156

Teng L, Lee S, Ginn A, Markland SM, Mir RA, DiLorenzo N, Boucher C, Prosperi M, Johnson J, Morris JG Jr *et al* (2019) Genomic comparison reveals natural occurrence of clinically relevant multidrug-resistant extended-spectrum-beta-lactamase-producing *Escherichia coli* strains. *Appl Environ Microbiol* 85: e03030-18

Teng L, Lee S, Park D, Jeong KC (2020) Genetic and functional analyses of virulence potential of an *Escherichia coli* O157:H7 strain isolated from super-shedder cattle. *Front Cell Infect Microbiol* 10: 271

Wang H, Jia C, Li H, Yin R, Chen J, Li Y, Yue M (2022) Paving the way for precise diagnostics of antimicrobial resistant bacteria. *Front Mol Biosci* 9: 976705

Wu S, Lu R, Zhang Y, Sun J (2010) Chronic *Salmonella* infected mouse model. *J Vis Exp* 39: e1947

Wu B, Ed-Dra A, Pan H, Dong C, Jia C, Yue M (2021) Genomic investigation of *Salmonella* isolates recovered from a pig slaughtering process in Hangzhou, China. *Front Microbiol* 12: 704636

Xu X, Chen Y, Pan H, Pang Z, Li F, Peng X, Ed-Dra A, Li Y, Yue M (2020) Genomic characterization of *Salmonella* uzaramo for human invasive infection. *Microb Genom* 6: mgen000401

Yue M, Song H, Bai L (2020) Call for special issue papers: food safety in China: current practices and future needs. *Foodborne Pathog Dis* 17: 295

Yue M, Bai L, Song H, Fang W (2021) Impacts of microbial food safety in China and beyond. *Foodborne Pathog Dis* 18: 508 – 509

Zhang S, den Bakker HC, Li S, Chen J, Dinsmore BA, Lane C, Lauer AC, Fields PI, Deng X (2019) SeqSero2: rapid and improved *Salmonella* serotype determination using whole-genome sequencing data. *Appl Environ Microbiol* 85: e01746-19

Zhou Z, Alikhan N-F, Mohamed K, Fan Y, Achtman M, Brown D, Chattaway M, Dallman T, Delahay R, Kornschober C (2020) The EnteroBase user's guide, with case studies on *Salmonella* transmissions, *Yersinia* pestis phylogeny, and *Escherichia* core genomic diversity. *Genome Res* 30: 138 – 152

License: This is an open access article under the terms of the Creative Commons Attribution License, which permits use, distribution and reproduction in any medium, provided the original work is properly cited.

Expanded View Figures

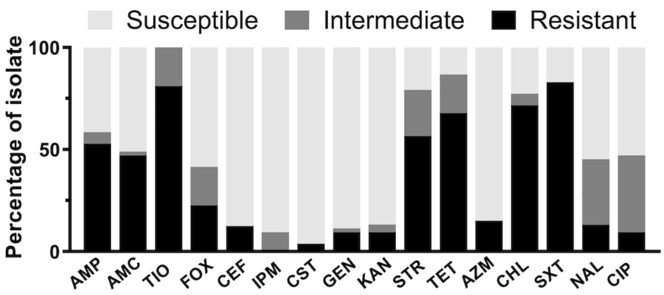

Figure EV1. Antimicrobial susceptibility testing.

A total of 53 SL isolates were tested for susceptibility to 16 antimicrobials in aerobic conditions, i.e. ampicillin (AMP), amoxicillin-clavulanic acid (AMC), ceftiofur (-TIO), cefoxitin (FOX), imipenem (IPM), gentamicin (-GEN), kanamycin (KAN), streptomycin (STR), tetracycline (TET), ciprofloxacin (CIP), nalidixic acid (NAL), trimethoprim-sulfamethoxazole (SXT), colistin (CST), azithromycin (AZM), ceftriaxone (CRO) and chloramphenicol (CHL). Cutoffs are listed in the source data file.

Source data are available online for this figure.

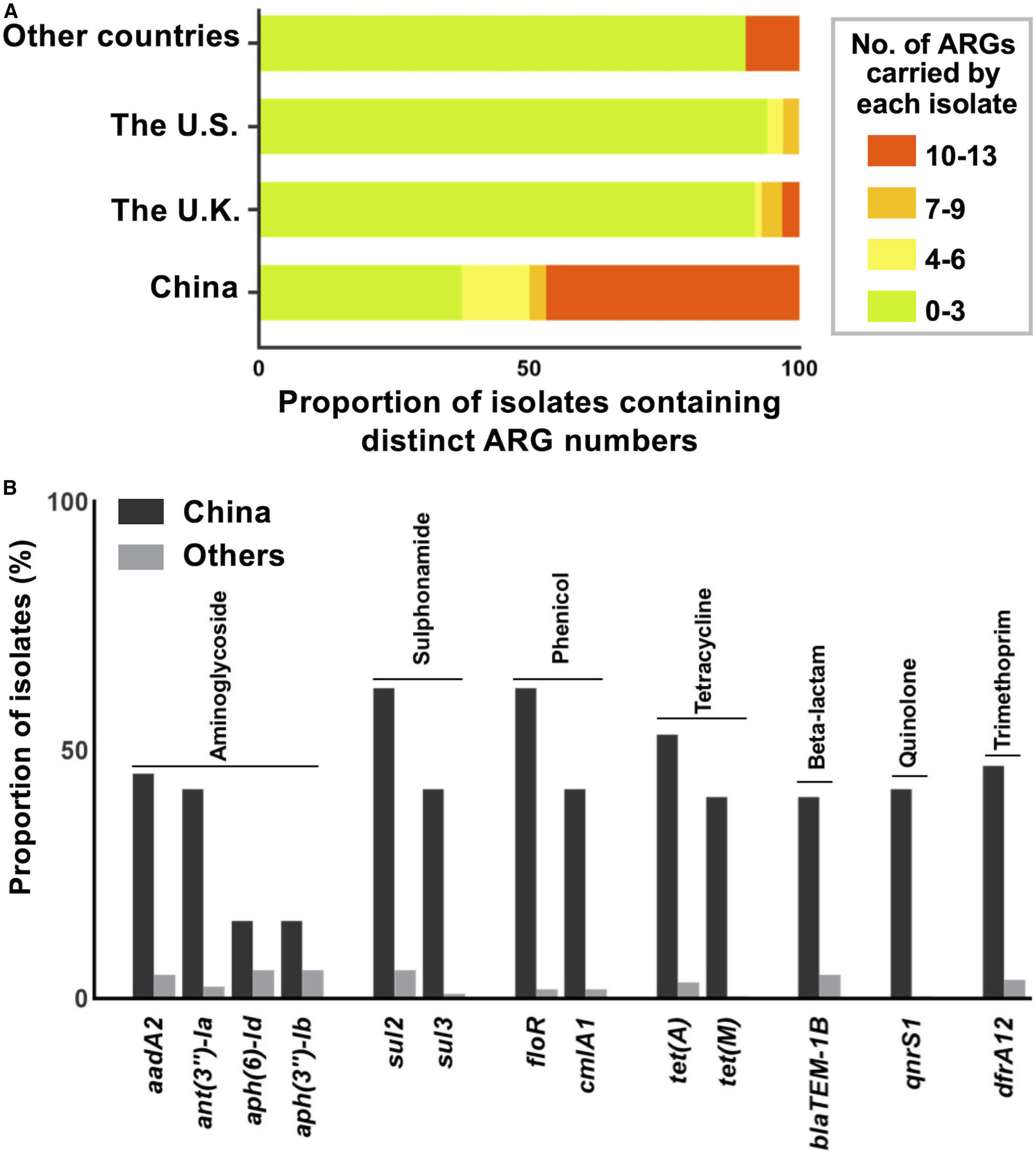

Figure EV2. Distribution of ARGs in SL isolates from China and other countries.

A Comparison of proportion of isolates containing distinct numbers of the antimicrobial-resistant gene from China and other countries.

B Proportion of ARGs in isolates from China and other countries. A total of 274 isolates were included in the analyses: 156 strains from the U.K.; 64 strains from China; 34 strains from the U.S.; 20 strains from other countries.

Source data are available online for this figure.

 ©2022 The Authors

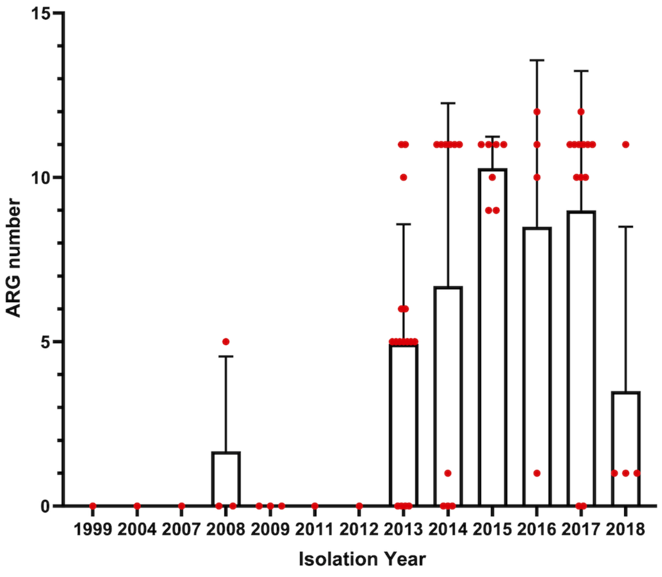

Figure EV3. The number of antimicrobial-resistant genes in isolates from distinct years.

The number of ARGs in all Chinese isolate were plotted against the isolation years. Red dots indicate the number of ARGs of each isolate. Data are presented as mean ± SD.

Source data are available online for this figure.

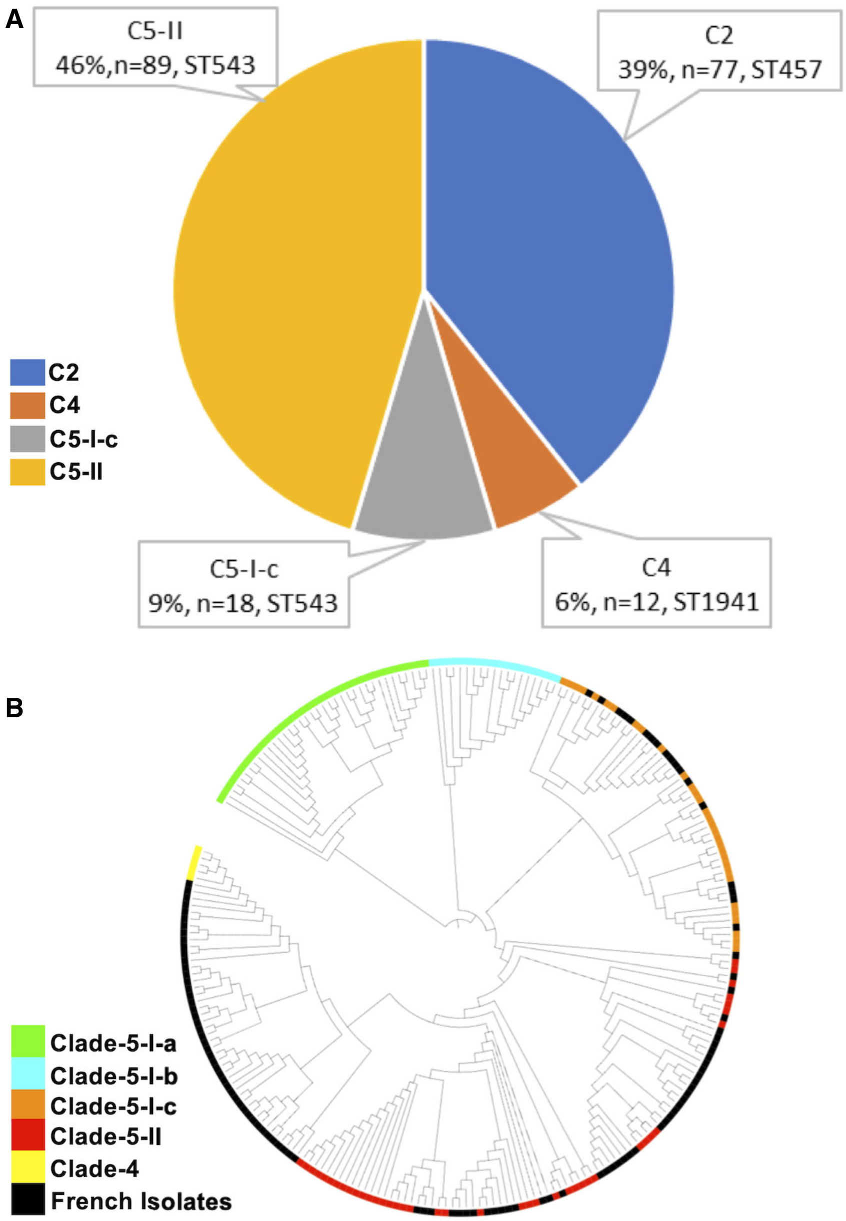

Figure EV4. Clade composition and phylogenetic tree of French isolates.

A The proportion of French isolates in each phylogenetic clade. The French isolates (n = 196) were from C2, C4, C5-I-c and C5-II.

B Phylogenetic relatedness of French and Chinese ST543 isolates. The French (n = 107) and Chinese ST543 isolates (n = 93) were used to construct a core-genome maximum-likelihood phylogenetic tree. The ring in different colours indicates the French isolates and distinct clades of Chinese isolates.

Source data are available online for this figure.

 ©2022 The Authors

Appendix Supplementary Methods

A non-typhoidal *Salmonella* serovar domestication accompanying enhanced niche adaptation

Table of content

**Table**	**Page**
Appendix Table S1. Conjugation frequency of *Salmonella* Livingstone isolates.	2
Appendix Figure S1. Growth curves of *S*. Livingstone isolates of 4 clades.	3
Appendix Figure S2. Survival of *C. elegans* challenged with *Salmonella* Livingstone.	4

Appendix Table S1. Conjugation frequency of *Salmonella* Livingstone isolates.

Name of isolates	Clade	Number of transconjugants (CFU)	Number of recipients (CFU)	Conjugation frequency
L12	C5-I-b	2.2×10^{6}	5.0×10^{6}	4.4×10^{-2}
L35	C5-I-b	1.4×10^{1}	3.0×10^{6}	4.7×10^{-6}
L41	C5-I-a	1.2×10^{2}	3.0×10^{6}	4.0×10^{-5}
L42	C5-I-a	1.6×10^{7}	8.0×10^{7}	2.0×10^{-2}
Lin-10	C5-I-a	2.0×10^{1}	1.0×10^{7}	2.0×10^{-6}

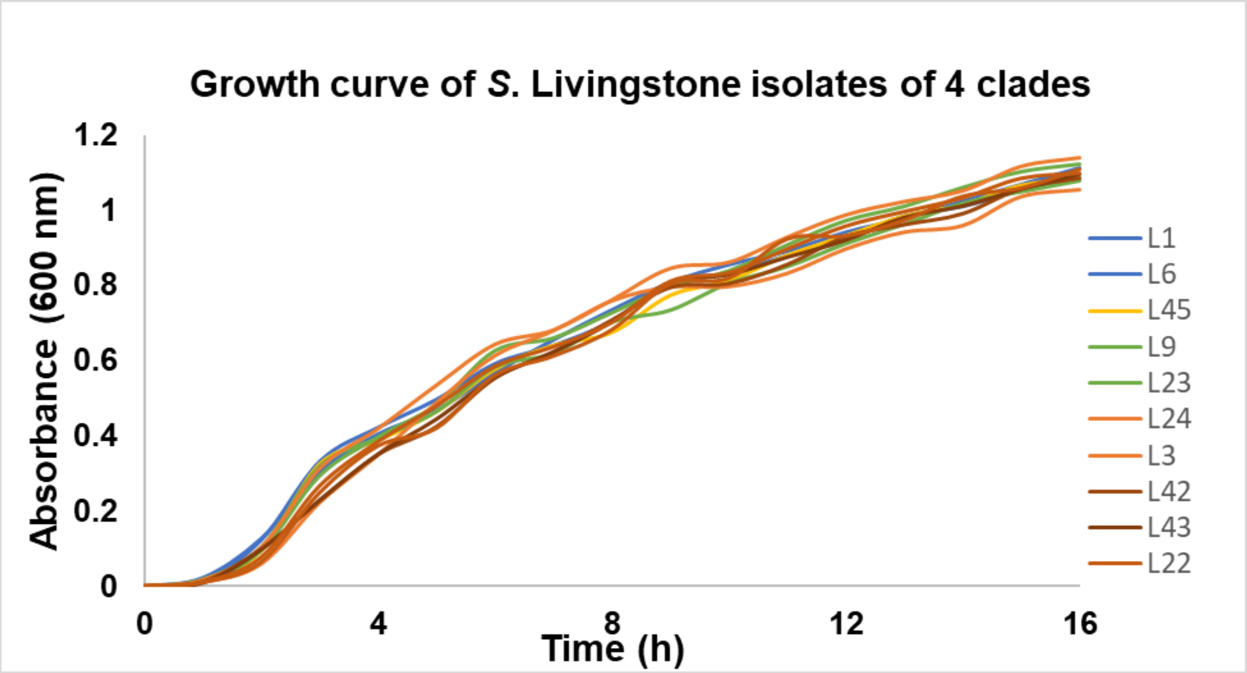

Appendix Figure S1. Growth curves of *S*. Livingstone isolates of 4 clades. The growth curves of the 10 isolates (Clade-1: strain L1 and L6; Clade-2: strain L45; Clade-3: strain L9 and L23; Clade-5: Clade-5-I-a strain L22, Clade-5-I-b strain L42 and L43, Clade-5-I-c strain L3 and L24) from 4 different clades were measured.

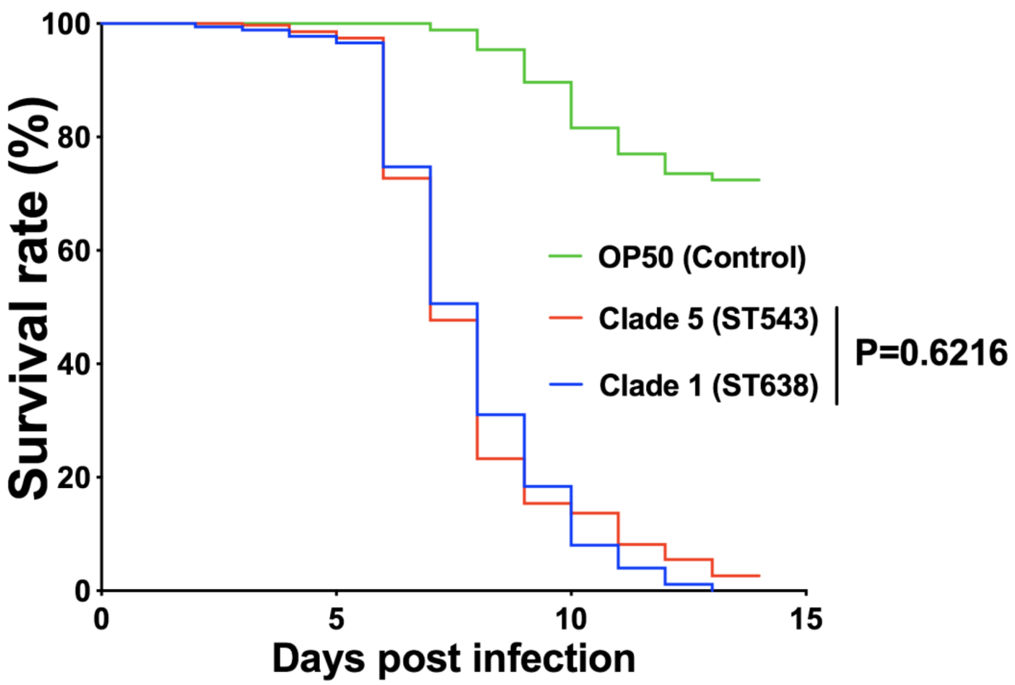

Appendix Figure S2. Survival of *C. elegans* challenged with *Salmonella* Livingstone. *C. elegans* was infected with isolates from Clade-1 and Clade-5 to evaluated their survival rates until day 14. The P value between two clades was calculated using Log-rank (Mantel-Cox, chi-square) test.
