# Supplementary material for: A nontyphoidal Salmonella serovar domestication accompanying enhanced niche adaptation
Source: EMBO Mol Med. 2022 Sep 29;14(11):e16366. doi: 10.15252/emmm.202216366 (PMC9641423; doi:10.15252/emmm.202216366)
Supplement: Supplementary file 13 [file EMMM-14-e16366-s007.pdf]

EMBO Molecular Medicine

# A non-typhoidal Salmonella serovar domestication accompanying enhanced niche adaptation

Yan Li, Lin Teng, Xuebin Xu, Xiaomeng Li, Xianqi Peng, Xiao Zhou, Jiaxin Du, Yanting Tang, Zhijie Jiang, Zining Wang, Chenghao Jia, Anja Müller, Corinna Kehrenberg, Haoqiu Wang, Beibei Wu, François-Xavier Weill, and Min Yue
**DOI: 10.15252/emmm.202216366**

*Corresponding author: Min Yue (myue@zju.edu.cn)*



## Review Timeline:

| | |
|---|---|
| Submission Date: | 24th May 22 |
| Editorial Decision: | 11th Jul 22 |
| Revision Received: | 16th Aug 22 |
| Editorial Decision: | 7th Sep 22 |
| Revision Received: | 9th Sep 22 |
| Accepted: | 12th Sep 22 |

*Editor: Zeljko Durdevic*


## Transaction Report:

(Note: With the exception of the correction of typographical or spelling errors that could be a source of ambiguity, letters and reports are not edited. Depending on transfer agreements, referee reports obtained elsewhere may or may not be included in this compilation. Referee reports are anonymous unless the Referee chooses to sign their reports.)

11th Jul 2022

Dear Prof. Yue,

Thank you for the submission of your manuscript to EMBO Molecular Medicine. Please accept my apologies for the unusual delay in getting back to you due to the longer time one referee required to complete evaluation of your manuscript.

We have now received feedback from the three reviewers who agreed to evaluate your manuscript. As you will see from the reports below, the referees acknowledge the interest of the study but also raise important and partially overlapping concerns that should be addressed in a major revision. Particularly, referees agree that the manuscript lacks focus, and that overwhelming amount of data detracts from the main finding that the clade 5-I-a/b is highly resistant and highly pathogenic as pointed out by the referee #1. Therefore, I would like you to reformat your manuscript as a scientific report (3 figures, ~22000 characters) for further consideration. For more information, please check our "Author Guidelines".
https://www.embopress.org/page/journal/17574684/authorguide#reportsarticleguide

Further consideration of a revision that addresses reviewers' concerns in full will entail a second round of review. EMBO Molecular Medicine encourages a single round of revision only and therefore, acceptance or rejection of the manuscript will depend on the completeness of your responses included in the next, final version of the manuscript. For this reason, and to save you from any frustrations in the end, I would strongly advise against returning an incomplete revision.

We would welcome the submission of a revised version within three months for further consideration. Please let us know if you require longer to complete the revision.

Please use this link to login to the manuscript system and submit your revision: Link Not Available

I look forward to receiving your revised manuscript.

Yours sincerely,

Zeljko Durdevic

Zeljko Durdevic
Editor
EMBO Molecular Medicine

*****

***** Reviewer's comments *****

Referee #1 (Comments on Novelty/Model System for Author):

multiple animal models used (C. elegans, chicken, zebrafish and mice) with a decent correlation between the results

Referee #1 (Remarks for Author):

the manuscript by Li et al. describes in detail a relatively understudied serovar of salmonella (S. Livingstone) from different angles. The most interesting, in my opinion, observation is that there is a significant within-serovar variability of the isolates in respect to the drug-resistance and invasiveness, which was relatively tightly associated with specific genomic clades. Such distinct phenotype/genotype variability has not been described that clearly within other serovars that include multi-drug resistant and invasive isolates. The invasive non-typhoidal salmonella is a very important and intriguing group of isolates, which is still poorly understood. Thus, identification the specific and highly phenotypically distinct clades in this serovar should provide important insights what make iNTS more pathogenic.

Major comments: the authors start with a description of five clinical isolates that caused systemic infections. However, those isolates are not mentioned virtually in any of the following results sections. For example, only by careful reading of the methods it is clear what clade the isolates belong to. Supplemental table 4 is the only figure/table (besides the original Table 1) where any additional info is provided about the strains. Information about those strains (both genotypic and phenotypic) should be provided across the results section.

Minor comments: I am not sure how much the description of aerobic/anaerobic conditions of the resistance adds without mechanistic insights. Same is about the biofilm formation.

Overall, the authors seemed to include too much data, which I find somewhat detractive from the most exciting finding that the clade 5-I-a/b is highly resistant and highly-pathogenic, and a result of recent adaptive evolution. The rest could be omitted or condensed.

Referee #2 (Remarks for Author):

This is a well written paper. The methods used are state of the art and scientifically sound.

Comments:

Line 64: Which standards (cut off's) were used to evaluate the results?

Line 79: there is too much about SARS-CoV-2; please focus on Salmonella

Line 96: if here "Salmonella enterica serovar Typhi", also change line 58 to: "Salmonella enterica serovar Livingstone"

Line 98: change "Numerous" to "numerous"

Line 129: is this "oxygen dependence" specific for S. Livingstone are also observed for other serovars?

Line 133: what's about the zebrafish experiments?

Line 158: change "conducted" to "provided"

Line 470: please delete the dot after China

Line 495: please delete "a" before significantly

Line 511: it would be interesting to have sopD2 and ratB mutants and to have the results in the animal models used in this study; the study mentioned in the discussion with S. Typhimurium has not used the same models; moreover, in contrast to S. Livingstone sopD2 and ratB are found in all S. Typhimurium

Line 525ff: What is the hypothesis for this observation (change in MIC's under anaerobic conditions)

Line 575: is there more evidence for this "animal feed import" argument (e.g. feed chain informations); the authors provide so far "only" results from molecular genetic comparisons of strains

Line 640: bla TEM is not responsible for a 3rd-generation cephalosporin resistance!

Line 706: there are coauthors in the author list without a declaration of their contributions

Line 826: Salmonella in italics

Line 839: Salmonella in italics

Line 862: Salmonella in italics

Line 864: Salmonella in italics

Line 866: Salmonella in italics

Line 868: Salmonella in italics

Line 871: Salmonella in italics

Line 876: Salmonella in italics

Line 907: E. coli in italics

Line 910: E. coli in italics

Line 932: mcr-1 and Salmonella in italics

Line 935: Salmonella in italics

Line 937: Salmonella in italics

Line 942: Salmonella in italics

Line 949: L. monocytogenes in italics

Line 953: Salmonella in italics

Line 976: Salmonella in italics

Line 897: Salmonella in italics

Line 1008: Salmonella in italics

Referee #3 (Remarks for Author):

The study aimed to examine the pathogenesis or invasiveness of Salmonella Livingstone. Phylogenetic analysis and antimicrobial resistance were also examined. The goals of the study appear too diverse. I would suggest to the authors to either include the outbreak isolates in the phenotypic and genomic analyses performed, or to strongly consider submitting a different manuscript describing the antimicrobial resistance study. The following points are provided to authors to improve the manuscript.
1. The conjugation efficiency of different clades was described, but what is the relevance of the result to the pathogenesis to resistance or virulence?
2. Animal studies cannot truly reflect the virulence potential (to humans) of the many different serovars of NTS. Although a higher pathogenicity of clade-5 was revealed from the animal experiments, there is no either clinical or epidemiological evidence indicating "the emergence of Chinese clade-5-I-a/b is more pathogenic for vertebrates".
3. The manuscript describes a lot of work done, but the authors fail in connecting the information. The methods are not consistently described (discrepancies between Methods and Supplementary data), and the different points made throughout the Discussion are therefore unconnected.

******* Reviewer's comments *******

**Referee #1**

(Comments on Novelty/Model System for Author):

multiple animal models used (C. elegans, chicken, zebrafish and mice) with a decent correlation between the results

Referee #1 (Remarks for Author):

the manuscript by Li et al. describes in detail a relatively understudied serovar of salmonella (S. Livingstone) from different angles. The most interesting, in my opinion, observation is that there is a significant within-serovar variability of the isolates in respect to the drug-resistance and invasiveness, which was relatively tightly associated with specific genomic clades. Such distinct phenotype/genotype variability has not been described that clearly within other serovars that include multi-drug resistant and invasive isolates. The invasive non-typhoidal salmonella is a very important and intriguing group of isolates, which is still poorly understood. Thus, identification the specific and highly phenotypically distinct clades in this serovar should provide important insights what make iNTS more pathogenic.

Thank you so much for your time and effort in reviewing our work. We believe that the iNTS in *Salmonella* serovars have broad implications, which could significantly improve the understanding of genetic diversity and accelerate public health surveillance in recognition of unknown threats, including their transmission and evolution history. We believe this is the central idea of this study.

Major comments: the authors start with a description of five clinical isolates that caused systemic infections. However, those isolates are not mentioned virtually in any of the following results sections. For example, only by careful reading of the methods it is clear what clade the isolates belong to. Supplemental table 4 is the only figure/table (besides the original Table 1) where any additional info is provided about the strains. Information about those strains (both genotypic and phenotypic) should be provided across the results section.

We highly appreciate the reviewer's helpful comments. We agree with the reviewer and added the genotypic and phenotypic information about the clinical strains across the results section. The details are listed here:

1) We added the ST information of the five clinical isolates in the **new Line 119-Line 120**: "**Notably, 5 iNTS strains belong to ST543 and C-5.**".

2) The result of antimicrobial susceptibility testing of those five iNTS isolates was emphasized: "**All five iNTS strains (L39, L42, L43, L44 and 18B003) were MDR and phenotypically resistant to more than 4 classes of antibiotics.** (**new Line 149-151**).

Also, we added more detailed information (i.e., Phylogenetic clade and antimicrobial-resistant profile) of those five clinical isolates into the new “**Table EV1. Clinical investigations of cases caused by invasive *Salmonella* Livingstone**.”

**Table EV1. Clinical investigations of cases caused by invasive *Salmonella* Livingstone.**

| Strain | Isolation Host | Age | Province (Isolation Year) | Isolation Source | Failure Antibiotic Treatment | Successful Antibiotic Treatment | Phylogenetic clade | Antimicrobial-resistant profile[a] |
|---|---|---|---|---|---|---|---|---|
| L42 | Human (Patient #1) | 4 months | Shanghai (2016) | Blood | Ceftriaxone (2 days), Azithromycin (3 days) | Imipenem (full course) | C5-I-b | AMP-AMC-TIO-FOX-CRO-GEN-KAN-STR-TET-AZM-CHL-SXT |
| L44 | Human (Patient #2) | 8 months | Fujian (2016) | Blood | Ceftriaxone (3 days), Azithromycin (3 days) | Imipenem (full course) | C5-I-a | AMP-AMC-TIO-STR-TET-AZM-CHL-SXT |
| L43 | Human (Patient #3) | 51 years | Shanghai (2016) | Blood | Ceftriaxone and Ciprofloxacin (5 days) | Colistin (full course) | C5-I-b | AMP-AMC-TIO-FOX-CRO-GEN-KAN-STR-TET-AZM-CHL-SXT |
| 18B003 | Human (Patient #4) | 45 years | Zhejiang (2019) | Blood | Azithromycin (5 days) | Ceftriaxone (full course) | C5-I-a | AMP-AMC-TIO-STR-TET-CHL-SXT-NAL |
| L39 | Human (Patient #5) | 1 year | Chongqing (2015) | Synovial fluid | | Ceftriaxone (full course) | C5-I-a | STR-TET-CHL-STX |

a. Abbreviations: ampicillin (AMP), amoxicillin-clavulanic acid (AMC), ceftiofur (TIO), cefoxitin (FOX), imipenem (IPM), gentamicin (GEN), kanamycin (KAN), streptomycin (STR), tetracycline (TET), colistin (CST), azithromycin (AZM), ceftriaxone (CRO), chloramphenicol (CHL), nalidixic acid (NAL), and trimethoprim-sulfamethoxazole (SXT).

3) The stress response of the iNTS isolates to oxidative stress, and desiccation stress was added to the **new Line 218-222** (“**The growth rates of isolates from four clades were similar without significant difference (Appendix Fig. S1), however, the C-5 (iNTS isolates L42 and L43) and C-4 isolates were significantly more resistant to hydrogen peroxide treatment than C-1 isolates (Fig. 3A), suggesting improved survivability within macrophages.**”).

The result of biofilm formation of the iNTS isolates was added to the **new Line 223-225** (“**The biofilm-forming capability of C-5 isolates is higher than that of C-1 and C-2 isolates but less than that of C-4 isolates (Fig. 3C).**”).

4) The animal infection results (i.e., chicken embryos, zebrafish embryos and mice) of the iNTS isolates were added to the **new Line 234-238,** i.e., **"C-5 iNTS isolates (e.g. L42 and L43) caused significantly higher deaths than C-1 isolates in chicken embryos at five days post-infection (dpi). Similarly, compared with C-1 isolates, iNTS isolates of C-5 lead to significantly higher number of zebrafish deaths (Fig. 3E)."**

and the **new Line 240-241** i.e., **"The bacterial load in the liver and cecum of infected mice was significantly higher for C-5 than that of C-1".**

Minor comments: I am not sure how much the description of aerobic/anaerobic conditions of the resistance adds without mechanistic insights. Same is about the biofilm formation.

Overall, the authors seemed to include too much data, which I find somewhat detractive from the most exciting finding that the clade 5-I-a/b is highly resistant and highly-pathogenic, and a result of recent adaptive evolution. The rest could be omitted or condensed.

1) We appreciate the constructive comments from the reviewer. We agree that we put too much data (i.e., description of aerobic/anaerobic conditions of the resistance) which is not directly related to our most exciting finding. **Hence, we removed the description of antimicrobial resistance in the anaerobic condition in the manuscript.**

2) We kept the result of antimicrobial susceptibility testing under aerobic condition because the enhanced antimicrobial resistance was accompanied by the evolution of the emerging clade 5-I-a/b clades. These results were rephrased and listed in the **new Line 145- Line 151**:

"**We evaluated the MIC of 53 Chinese SL isolates against 16 antibiotics. More than half of the isolates were resistant or intermediate to ceftiofur (100%), tetracycline (86.8%), sulfamethoxazole-trimethoprim (71.7%), streptomycin (79.2%), chloramphenicol (77.4%) and ampicillin (58.5%) (Fig. EV1; Table EV1). In total, 51 out of 53 isolates were MDR. All five iNTS strains (L39, L42, L43, L44 and 18B003) were MDR and phenotypically resistant to more than 4 classes of antibiotics".**

3) However, we believe that the information on biofilm formation is necessary and should be kept in the manuscript. Here is the rationale: bacterial biofilms are their "protective clothing" in extreme environments, therefore a good biofilm formation capability can enhance their survivability in harsh environments, contributing to their adaptation to niches and pandemic.

The result of biofilm assay as kept in the **new Line 223-225**: "**The biofilm-forming capability of C-5 isolates is higher than that of C-1 and C-2 isolates but less than that of C-4 isolates (Fig. 3C).**"

**Referee #2 (Remarks for Author):**

This is a well written paper. The methods used are state of the art and scientifically sound.

Thanks so much for your time and effort in improving our work.

Comments:

Line 64: Which standards (cut off's) were used to evaluate the results?

Thank you for the comments. The standards (cutoffs) were from CLSI, NCCLS and NARMS (i.e., National Antimicrobial Resistance Monitoring System for Enteric Bacteria).

1) We added the description of the cutoffs in the Materials and Methods section in the **new Line 354-361: "Minimal inhibitory concentrations (MIC) were determined by the broth microdilution assay, according to the criteria recommended by the Clinical and Laboratory Standards Institute (CLSI) and National Antimicrobial Resistance Monitoring System for Enteric Bacteria (NARMS) (https://www.cdc.gov/narms/antibiotics-tested.html) (Table S1) (CLSI, 2016). Except for ceftiofur and streptomycin, the breakpoints for the antibiotics were from CLSI. The breakpoints for ceftiofur and streptomycin were from NCCLS and NARMS, respectively.".**

2) Besides, to make the cutoffs clear, we listed them in the **new** "**Source data for EV1**".

**Source Data for Fig EV1. Criteria of MIC**

| **Antibiotic class** | **Antimicrobials** | **Range of antibiotic concentration (μg/ml)** | **Clinical breakpoints [b] (μg/ml)** | **No. of resistant and intermediate strains and percentage** |
|---|---|---|---|---|
| Beta-lactams | TIO | 1-128 | ≥8 [c] | 53 (100.0%) |
| | FOX | 0.5-64 | ≥32 | 22 (41.5%) |

| | | | | |
|---|---|---|---|---|
| | CRO | 0.5-64 | ≥4 | 6 (11.3%) |
| | AMP | 0.5-64 | ≥32 | 31 (58.5%) |
| | AMC | 0.5-64 | ≥32/16 | 26 (49.1%) |
| | IPM | 0.125-64 | ≥4 | 5 (9.4%) |
| Tetracyclines | TET | 0.5-64 | ≥16 | 46 (86.8%) |
| Antifolates | SXT | 0.25-32 | ≥4/76 | 38 (71.7%) |
| Aminoglycosides | STR | 0.5-64 | ≥32 [d] | 42 (79.2%) |
| | GEN | 0.25-32 | ≥16 | 6 (11.3%) |
| | KAN | 0.5-64 | ≥64 | 7 (13.2%) |
| Phenicols | CHL | 0.5-64 | ≥32 | 41 (77.4%) |
| Quinolones | CIP | 0.125-16 | ≥1 | 25 (47.2%) |
| | NAL | 0.5-64 | ≥32 | 24 (45.3%) |
| Macrolides | AZM | 0.5-64 | ≥32 | 8 (15.1%) |
| Lipopeptides | CST | 0.0625-32 | ≥4 | 2 (3.8 %) |

a. Abbreviations: ampicillin (AMP), amoxicillin-clavulanic acid (AMC), ceftiofur (TIO), cefoxitin (FOX), imipenem (IPM), gentamicin (GEN), kanamycin (KAN), streptomycin (STR), tetracycline (TET), ciprofloxacin (CIP), nalidixic acid (NAL), trimethoprim-sulfamethoxazole (SXT), colistin (CST), azithromycin (AZM), ceftriaxone (CRO) and chloramphenicol (CHL).

b. The breakpoints are from CLSI unless otherwise stated. (CLSI. Performance Standards for Antimicrobial Susceptibility Testing. 30th ed. CLSI supplement M100. Wayne, PA: Clinical and Laboratory Standards Institute; 2020.)

c. The breakpoint for ceftiofur is from NCCLS. (NCCLS. Performance Standards for Antimicrobial Disk and Dilution Susceptibility Tests for Bacteria Isolated from Animals; Approved Standard—Second Edition. NCCLS document M31-A22 [ISBN 1-56238-461-9]. NCCLS, 940 West Valley Road, Suite 1400, Wayne, Pennsylvania 19087-1898 USA, 2002.)

d. The breakpoint for streptomycin is established by NARMS. Website: https://www.cdc.gov/narms/antibiotics-tested.html

Line 79: there is too much about SARS-CoV-2; please focus on Salmonella

Thank you for the suggestion. We agree with your comment and removed the contents of SARS-CoV-2 in the introduction section properly.

Line 96: if here "Salmonella enterica serovar Typhi", also change line 58 to: "Salmonella enterica serovar Livingstone"

Thank you for the comment. We used “*Salmonella enterica* serovar Livingstone” to replace the original description (**new Line 57**).

Line 98: change "Numerous" to "numerous"

We appreciate the comment. When we revised the manuscript accordingly, this sentence was removed so no more change is needed.

Line 129: is this "oxygen dependence" specific for S. Livingstone are also observed for other serovars?

This "oxygen dependence" is also observed in other *Salmonella* serovars. In our unpublished data, we found the "oxygen dependence" in *Salmonella* Gallinarum and *Salmonella* Pullorum.

The result of "oxygen dependence" is removed in the revised manuscript based on the suggestions by the other two reviewers.

Line 133: what's about the zebrafish experiments?

Thank you for the comment. The ethical statements for zebrafish experiments were added in the **new Line 330-333**: **"The research protocol for mice, chicken and zebrafish infection experiments conducted in this study was approved by the Committee of Laboratory Animal Center of Zhejiang University (ZJU20190094 and ZJU20170360)."**

Line 158: change "conducted" to "provided"

Thank you for the reviewer's correction. "provided" was added to replace "conducted" **(new Line 372).**

Line 470: please delete the dot after China

We appreciated the correction by the reviewer. The dot after China was deleted **(new Line 215): "Nevertheless, unique clones, C-5-I-a/b, were primarily found in China."**

Line 495: please delete "a" before significantly

Thank you for the correction. The "a" was deleted. **(new Line 196)**

Line 511: it would be interesting to have sopD2 and ratB mutants and to have the results in the animal models used in this study; the study mentioned in the discussion with S. Typhimurium has not used the same models; moreover, in contrast to S. Livingstone sopD2 and ratB are found in all S. Typhimurium

We appreciate the helpful comments from the reviewer. We have tried to get the *sopD2* and *ratB* mutants of Clade-5 Chinese isolates, however, all these Clade-5 isolates carrying these two genes are multidrug-resistant (extensive resistance towards commonly used six antibiotic classes). Thus, we could not use the traditional antibiotic-based approach to make the mutants to test this hypothesis. We believe this could be an interesting point for further investigation in the future.

Besides, we rewrote the discussion section of these two genes **(new Line 302-305)** based on your comments: **"Besides, Kinsley et al., demonstrated the *ratB***

**deletion mutant of *S.* Typhimurium showed defect for colonization of the mice cecum (Kingsley, Humphries et al., 2003). Further study is needed for understanding clade- or subclade-specific virulence."**

Line 525: What is the hypothesis for this observation (change in MIC's under anaerobic conditions)

The clinical iNTS Livingstone strains were mostly (4 out of 5 strains) associated with antimicrobial treatment failure. Since the intestinal tract of humans contains low oxygen concentration, we hypothesize that low oxygen concentration (or anaerobic condition) affects the MIC of antibiotics, resulting in antimicrobial treatment failure.

The other two reviewers mentioned that the overwhelming amount of data detracts from the main finding that the clade 5-I-a/b is highly resistant and highly pathogenic. They suggested deleting the results of MIC under anaerobic conditions. Therefore, we followed their comments to remove the result of the anaerobic MIC assay and focused on the emergence of clade 5-I-a/b.

Line 575: is there more evidence for this "animal feed import" argument (e.g. feed chain informations); the authors provide so far "only" results from molecular genetic comparisons of strains

We observed that the *Salmonella* isolates, including *S*. Livingstone were frequently identified from the imported animal feeds (a kind of unexpected phenomenon). Apart from the results from molecular genetic comparisons of strains, we also provide additional evidence from the literature to support our findings and add them to the discussion section.

For example: (**new Line 272-280) "Notably, the detection of SL in fish meals imported to China was reported in 1990s (Chen, 1994), and the amount of imported fish meals increased 90 times during past 4 decades (IndexMundi, 2022), highlighting that the fish meals is a transfer vehicle for SL spreading. In support of international feed-borne transmission, a recent study demonstrated the strong survivability of SL in a low-moisture environment (Abdelhamid & Yousef, 2021), in consistence with high resistance to desiccation (fish meal environments) for our fish meals-derived C-1 isolates (isolate L1 from fish meal imported to China, Fig. 3)"**

In addition to the above evidence, many studies also observed the presence of *Salmonella* Livingstone and other *Salmonella* serotypes in low moisture environments, such as animal feed. Nabbut et al., and Mårtensson et al., found the occurrence of *Salmonella* in animal feed, indicating animal feed can be a source of *Salmonella* (Nabbut NH, Barbour EK, Al-Nakhli HM. Occurrence of salmonellae in animal feed ingredients in Saudi Arabia. Am J Vet Res. 1982 Sep;43(9):1703-5.; Mårtensson L, Holmberg T, Hurvell B, Rutqvist L, Sandstedt K, Wierup M. Salmonella isolated from animals and feed stuffs in Sweden during 1978-1982. Nord Vet Med. 1984 Nov-Dec;36(11):371-93. PMID: 6531210.). Abdelhamid et al., identified desiccation-resistant *Salmonella* Livingstone in low-moisture food. (Abdelhamid AG, Xu Y, Yousef AE. Draft

Genome Sequence of *Salmonella enterica* subsp. enterica Serovar Livingstone 1236H, a Desiccation-Resistant Strain That Poses a Salmonellosis Hazard in Low-Moisture Foods. Microbiol Resour Announc. 2021 Jan 7;10(1):e01197-20. doi: 10.1128/MRA.01197-20.). After the animals (e.g., chicken) ate feed contaminated by *Salmonella*, they will be able to get an infection (Hinton M, Salmonella infection in chicks following the consumption of artificially contaminated feed. Epidemiol Infect. 1988 Apr;100(2):247-56. doi: 10.1017/s0950268800067388.). Taken together, animal feed imported to China could serve as a source of this particular pathogen.

Line 640: bla TEM is not responsible for a 3rd-generation cephalosporin resistance!

Thank you for the correction. The “blaTEM” is removed **(new Line 316).**

Line 706: there are coauthors in the author list without a declaration of their contributions

Thank you for the correction. We added all the coauthors and their contributions in “Author contribution” section in the revised manuscript **(new Line 552-568)**:

“Yan Li: Conceptualization; resources; data curation; validation; investigation; methodology; supervision; funding acquisition, writing – review and editing. Lin Teng: Conceptualization; data curation; validation; investigation; formal analysis, visualization; methodology; writing – original draft; writing – review and editing. Xuebin Xu: Conceptualization; resources; data curation, writing – review and editing. Xiaomeng Li: Data curation; validation; investigation; visualization; methodology; writing – original draft. Xianqi Peng: Investigation; Methodology. Xiao Zhou: Investigation; Methodology. Jiaxin Du: Investigation; Methodology. Yanting Tang: Investigation; Methodology. Zhijie Jiang: Investigation; Methodology. Zining Wang: Investigation; Methodology. Chenghao Jia: Investigation; Methodology. Anja Müller: Investigation, writing – review and editing. Corinna Kehrenberg: Investigation, writing – review and editing. Haoqiu Wang: Resources; data curation. Beibei Wu: Resources; data curation. François-Xavier Weill: Resources; investigation, writing – review and editing. Min Yue: Conceptualization; resources; data curation; validation; investigation; supervision; funding acquisition, writing – review and editing; and project administration.”

Line 826: Salmonella in italics

All “*Salmonella*” in the references were changed in italics.

Line 839: Salmonella in italics

All “*Salmonella*” in the references were changed in italics.

Line 862: Salmonella in italics

All “*Salmonella*” in the references were changed in italics.

Line 864: Salmonella in italics

All "*Salmonella*" in the references were changed in italics.

Line 866: Salmonella in italics

All "*Salmonella*" in the references were changed in italics.

Line 868: Salmonella in italics

All "*Salmonella*" in the references were changed in italics.

Line 871: Salmonella in italics

All "*Salmonella*" in the references were changed in italics.

Line 876: Salmonella in italics

All "*Salmonella*" in the references were changed in italics.

Line 907: E. coli in italics

All "*E. coli*" in the references section were changed in italics.

Line 910: E. coli in italics

The "*E. coli*" in the references section were changed in italics.

Line 932: mcr-1 and Salmonella in italics

The "*mcr-1* and *Salmonella*" were changed in italics.

Line 935: Salmonella in italics

All "*Salmonella*" in the references were changed in italics.

Line 937: Salmonella in italics

All "*Salmonella*" in the references were changed in italics.

Line 942: Salmonella in italics

All "*Salmonella*" in the references were changed in italics.

Line 949: L. monocytogenes in italics

The "*L. monocytogenes*" in the references were changed in italics.

Line 953: Salmonella in italics

All "*Salmonella*" in the references were changed in italics.

Line 976: Salmonella in italics

All "*Salmonella*" in the references were changed in italics.

Line 897: Salmonella in italics

All “*Salmonella*” in the references were changed in italics.

Line 1008: Salmonella in italics

All “*Salmonella*” in the references were changed in italics.

**Referee #3 (Remarks for Author):**

The study aimed to examine the pathogenesis or invasiveness of Salmonella Livingstone. Phylogenetic analysis and antimicrobial resistance were also examined.

The goals of the study appear too diverse.

I would suggest to the authors to either include the outbreak isolates in the phenotypic and genomic analyses performed, or to strongly consider submitting a different manuscript describing the antimicrobial resistance study. The following points are provided to authors to improve the manuscript.

We highly appreciate your insightful comments and suggestions, and completely agree that “the goals of the study appear too diverse”. Therefore, we removed the results of the antimicrobial resistance study and focused on the phenotypic and genomic characteristics of the outbreak isolates and the two novel clades in China.

1. The conjugation efficiency of different clades was described, but what is the relevance of the result to the pathogenesis to resistance or virulence?

Thank you for the reviewer’s comments. Given the isolates from two novel Chinese subclades simultaneously carried abundant ARGs and plasmid replicon genes, we hypothesized the conjugative transfer of these plasmids contribute to the acquisition of ARGs.

2. Animal studies cannot truly reflect the virulence potential (to humans) of the many different serovars of NTS. Although a higher pathogenicity of clade-5 was revealed from the animal experiments, there is no either clinical or epidemiological evidence indicating "the emergence of Chinese clade-5-I-a/b is more pathogenic for vertebrates"

Thanks for your comments. We agree that animal studies cannot truly reflect the virulence potential to humans and included such statement in the discussion and indicated that, though the five clinical isolates of Clade-5-I-a/b caused bacteremia and, thus, were invasive by definition, more extended clinical and/or epidemiological studies in both humans and animals need to be collected to establish the indicating that the clade-5-I-a/b is more pathogenic for vertebrates. Therefore, we rephrased the related sentences: “we found that the emerging Chinese C-5-I-a/b **might be** more virulent for vertebrates” (**new Line 243-244**).

Also, in the **new Line 264-267** (Discussion section), we used “chicken, zebrafish and mice” to replace “vertebrates”. Afterwards, “vertebrates” is not mentioned, and the new sentence is that “we observed the emerging MDR and hypervirulent subclades of SL with an acquired ARG-carrying plasmid and niche-specific adaptation evidenced by increased pathogenicity towards **chicken, zebrafish and mice**.”

3. The manuscript describes a lot of work done, but the authors fail in connecting the information. The methods are not consistently described (discrepancies between Methods and Supplementary data), and the different points made throughout the Discussion are therefore unconnected.

We appreciate the constructive comment from the reviewer. In the new version of our manuscript, we significantly revised the manuscript to connect all our results in a logical manner. Here is how we improve this manuscript:

1) Abstract:
The Abstract was modified:

**New Line 54-55**, “**However, the mechanism by which iNTS emerged in China remains unaddressed.**”

**New Line 64-66**, “**Overall, we offer exploiting genomics in uncovering international transmission led by the animal feed trade and highlight an emerging hypervirulent clade with increased resistance to frontline antibiotics.”**

2) Introduction section:

We modified the introduction section to focus on *Salmonella* by removing the descriptions not related to *Salmonella* (**new Line70-79, and new Line 82-86**).

3) Result section:

The results of the comparison of antimicrobial resistance under aerobic and anaerobic conditions were also removed. Also, we removed the result of the accuracy of WGS prediction, considering it is out of the scope of this study.

Based on the editor’s suggestion, we formatted this manuscript into a Report (a short article). Therefore, we shrank the description of the clinical cases to make the result more concise (**new Line 104-108**).

The results of phenotypic and genomic analyses of the iNTS isolates were mentioned in each section of the results. For example:

**New Line 119-120**: “Notably, five clinical iNTS strains belong to ST543 and C-5.”

**New Line 149-151:** “All five iNTS strains (L39, L42, L43, L44 and 18B003) were MDR and phenotypically resistant to more than 4 classes of antibiotics.”

**New Line 166-167**: “Notably, 5 iNTS isolates belong to the emerging C-5-I-a and C-5-I-b (Table EV1).”

**New Line 218-222**:” The growth rates of isolates from four clades were similar without significant difference (Appendix Fig. S1), however, the C-5 (iNTS isolates L42 and L43) and C-4 isolates were significantly more resistant to hydrogen peroxide treatment than C-1 isolates (Fig. 3A), suggesting improved survivability within macrophages.”

**New Line 224-226**: “The biofilm-forming capability of C-5 isolates is higher than that of C-1 and C-2 isolates but less than that of C-4 isolates (Fig. 3C).”

**New Line 236-239: “**C-5 iNTS isolates (e.g. L42 and L43) caused significantly higher deaths than C-1 isolates in chicken embryos at five days post-infection (dpi). Similarly, compared with C-1 isolates, iNTS isolates of C-5 lead to significantly higher number of zebrafish deaths (Fig. 3E).”

**New Line 240-241: “**The bacterial load in the liver and cecum of infected mice was significantly higher for C-5 than that of C-1,”

4) Discussion Section:

We reorganized and, further rewrote, the discussion section. In the first paragraph of the discussion, we summarized the major findings of our study. Then, we discussed the sources of the clinical *S*. Livingstone isolates in China in the second paragraph, following the discussion of bacterial evolution (i.e., enhanced virulence and accumulation of antimicrobial resistance) in the 3$^{rd}$ and 4$^{th}$ paragraphs. We discussed the potential reasons for the enhanced virulence of the Chinese Clade-5 isolates in the 3$^{rd}$ paragraph. The potential reasons for the acquisition of antimicrobial resistance of the novel clades were discussed in the 4$^{th}$ paragraph.

5) Materials and methods:

In the revised manuscript, we made Methods and Supplementary data consistent by adding and matching detailed information on methods and Supplementary data (EV figures, EV tables, and appendix). For instance: we added the growth curves results of isolates from distinct clades to the Appendix figure S1. Additionally, we put the detailed methods of desiccation assay and biofilm assay to the **new Line 463-475**). All the methods of statistical analyses were included in the Method section (**new Line 461-462, 474-475, 485, 507-508, 520-521, and 533-535**).

7th Sep 2022

Dear Prof. Yue,

Thank you for the submission of your revised manuscript to EMBO Molecular Medicine. I am pleased to inform you that we will be able to accept your manuscript pending the following final amendments:

1) In the main manuscript file, please do the following:
- Correct/answer the track changes suggested by our data editors by working from the attached document.
- Limit keywords to max. 5.
- Figures should be called out in a sequential order. Currently Figure 2B is called out after Figue 3 and Figure EV2B after Figure EV3. Please correct.
- In M&M, add statistical paragraph that should reflect all information that you have filled in the Authors Checklist, especially regarding randomization, blinding, replication.
- Correct the reference citation in the reference list. Citations should be listed in alphabetical order. Where there are more than 10 authors on a paper, 10 will be listed, followed by "et al.". Please check "Author Guidelines" for more information.
https://www.embopress.org/page/journal/17574684/authorguide#referencesformat
2) Appendix: Please rename Appendix Tables S1, S2, S4 and S5 to Datasets EV1-4 and Appendix Table S3 to Appendix Table S1. Also, update Appendix ToC and table callouts in the main text accordingly.
3) Funding: Please make sure that information about all sources of funding are complete in both our submission system and in the manuscript.
4) Author contributions: Please specify author contributions in our submission system. CRediT has replaced the traditional author contributions section because it offers a systematic machine readable author contributions format that allows for more effective research assessment. You are encouraged to use the free text boxes beneath each contributing author's name to add specific details on the author's contribution. More information is available in our guide to authors:
https://www.embopress.org/page/journal/17574684/authorguide#authorshipguidelines
5) Synopsis: Every published paper now includes a 'Synopsis' to further enhance discoverability. Synopses are displayed on the journal webpage and are freely accessible to all readers. They include separate synopsis image and synopsis text.
- Synopsis image: Please simplify provided visual abstract and submit it as a high-resolution jpeg file 550 px-wide x (250-400)-px high.
- Synopsis text: Please provide a short standfirst (maximum of 300 characters, including space) as well as 2-5 one sentence bullet points that summarise the paper as a .doc file. Please write the bullet points to summarise the key NEW findings. They should be designed to be complementary to the abstract - i.e. not repeat the same text. We encourage inclusion of key acronyms and quantitative information (maximum of 30 words / bullet point). Please use the passive voice.
- Please check your synopsis text and image before submission with your revised manuscript. Please be aware that in the proof stage minor corrections only are allowed (e.g., typos).
6) For more information: This space should be used to list relevant web links for further consultation by our readers. Could you identify some relevant ones and provide such information as well? Some examples are patient associations, relevant databases, OMIM/proteins/genes links, author's websites, etc...
7) As part of the EMBO Publications transparent editorial process initiative (see our Editorial at http://embomolmed.embopress.org/content/2/9/329), EMBO Molecular Medicine will publish online a Review Process File (RPF) to accompany accepted manuscripts. This file will be published in conjunction with your paper and will include the anonymous referee reports, your point-by-point response and all pertinent correspondence relating to the manuscript. Let us know whether you agree with the publication of the RPF and as here, if you want to remove or not any figures from it prior to publication. Please note that the Authors checklist will be published at the end of the RPF.
8) Please provide a point-by-point letter INCLUDING my comments as well as the reviewer's reports and your detailed responses (as Word file).

I look forward to reading a new revised version of your manuscript as soon as possible.

Yours sincerely,

Zeljko Durdevic

Zeljko Durdevic
Editor
EMBO Molecular Medicine

***** Reviewer's comments *****

Referee #1 (Comments on Novelty/Model System for Author):

there are no good models to test for salmonella's virulence. But they did what could be done in full

Referee #1 (Remarks for Author):

the authors did a really good job in reformatting and focusing the study. this is a much better scientific contribution now

Referee #2 (Remarks for Author):

The authors have considered the comments. The revision is adequate. No further comments.

The authors performed the requested editorial changes.

We are pleased to inform you that your manuscript is accepted for publication and is now being sent to our publisher to be included in the next available issue of EMBO Molecular Medicine.

## EMBO Press Author Checklist


| | |
|---|---|
| Corresponding Author Name: Min Yue | |
| Journal Submitted to: EMBO Molecular Medicine | |
| Manuscript Number: EMM-2022-16366 | |


**USEFUL LINKS FOR COMPLETING THIS FORM**
[The EMBO Journal - Author Guidelines]
[EMBO Reports - Author Guidelines]
[Molecular Systems Biology - Author Guidelines]
[EMBO Molecular Medicine - Author Guidelines]

**Reporting Checklist for Life Science Articles (updated January 2022)**
This checklist is adapted from Materials Design Analysis Reporting (MDAR) Checklist for Authors. MDAR establishes a minimum set of requirements in transparent reporting in the life sciences (see Statement of Task: 10.31222/osf.io/9sm4x). Please follow the journal's guidelines in preparing your manuscript.
**Please note that a copy of this checklist will be published alongside your article.**

**Abridged guidelines for figures**
**1. Data**
The data shown in figures should satisfy the following conditions:
- the data were obtained and processed according to the field's best practice and are presented to reflect the results of the experiments in an accurate and unbiased manner.
- ideally, figure panels should include only measurements that are directly comparable to each other and obtained with the same assay.
- plots include clearly labeled error bars for independent experiments and sample sizes. Unless justified, error bars should not be shown for technical replicates.
- if n<5, the individual data points from each experiment should be plotted. Any statistical test employed should be justified.
- Source Data should be included to report the data underlying figures according to the guidelines set out in the authorship guidelines on Data Presentation.

**2. Captions**
Each figure caption should contain the following information, for each panel where they are relevant:
- a specification of the experimental system investigated (eg cell line, species name).
- the assay(s) and method(s) used to carry out the reported observations and measurements.
- an explicit mention of the biological and chemical entity(ies) that are being measured.
- an explicit mention of the biological and chemical entity(ies) that are altered/varied/perturbed in a controlled manner.
- the exact sample size (n) for each experimental group/condition, given as a number, not a range;
- a description of the sample collection allowing the reader to understand whether the samples represent technical or biological replicates (including how many animals, litters, cultures, etc.).
- a statement of how many times the experiment shown was independently replicated in the laboratory.
- definitions of statistical methods and measures:
  - common tests, such as t-test (please specify whether paired vs. unpaired), simple χ2 tests, Wilcoxon and Mann-Whitney tests, can be unambiguously identified by name only, but more complex techniques should be described in the methods section;
  - are tests one-sided or two-sided?
  - are there adjustments for multiple comparisons?
  - exact statistical test results, e.g., P values = x but not P values < x;
  - definition of 'center values' as median or average;
  - definition of error bars as s.d. or s.e.m.

**Please complete ALL of the questions below.**
**Select "Not Applicable" only when the requested information is not relevant for your study.**

**Materials**

| Newly Created Materials | Information included in the manuscript? | In which section is the information available? (Reagents and Tools Table, Materials and Methods, Figures, Data Availability Section) |
|---|---|---|
| New materials and reagents need to be available; do any restrictions apply? | Not Applicable | |

| Antibodies | Information included in the manuscript? | In which section is the information available? (Reagents and Tools Table, Materials and Methods, Figures, Data Availability Section) |
|---|---|---|
| For **antibodies** provide the following information:<br>- Commercial antibodies: RRID (if possible) or supplier name, catalogue number and or/clone number<br>- Non-commercial: RRID or citation | Not Applicable | |

| DNA and RNA sequences | Information included in the manuscript? | In which section is the information available? (Reagents and Tools Table, Materials and Methods, Figures, Data Availability Section) |
|---|---|---|
| **Short novel DNA or RNA including primers, probes**: provide the sequences. | Not Applicable | |

| Cell materials | Information included in the manuscript? | In which section is the information available? (Reagents and Tools Table, Materials and Methods, Figures, Data Availability Section) |
|---|---|---|
| **Cell lines:** Provide species information, strain. Provide accession number in repository **OR** supplier name, catalog number, clone number, and/**OR** RRID. | Not Applicable | |
| **Primary cultures:** Provide species, strain, sex of origin, genetic modification status. | Not Applicable | |
| Report if the cell lines were recently **authenticated** (e.g., by STR profiling) and tested for mycoplasma contamination. | Not Applicable | |

| Experimental animals | Information included in the manuscript? | In which section is the information available? (Reagents and Tools Table, Materials and Methods, Figures, Data Availability Section) |
|---|---|---|
| **Laboratory animals or Model organisms:** Provide species, strain, sex, age, genetic modification status. Provide accession number in repository **OR** supplier name, catalog number, clone number, **OR** RRID. | Yes | Materials and Methods |
| **Animal observed in or captured from the field:** Provide species, sex, and age where possible. | Not Applicable | |
| Please detail **housing and husbandry conditions**. | Yes | Materials and Methods |

| Plants and microbes | Information included in the manuscript? | In which section is the information available? (Reagents and Tools Table, Materials and Methods, Figures, Data Availability Section) |
|---|---|---|
| **Plants:** provide species and strain, ecotype and cultivar where relevant, unique accession number if available, and source (including location for collected wild specimens). | Not Applicable | |
| **Microbes:** provide species and strain, unique accession number if available, and source. | Yes | Materials and Methods |

| Human research participants | Information included in the manuscript? | In which section is the information available? (Reagents and Tools Table, Materials and Methods, Figures, Data Availability Section) |
|---|---|---|
| If collected and within the bounds of privacy constraints report on age, sex and gender or ethnicity for all study participants. | Not Applicable | |

| Core facilities | Information included in the manuscript? | In which section is the information available? (Reagents and Tools Table, Materials and Methods, Figures, Data Availability Section) |
|---|---|---|
| If your work benefited from core facilities, was their service mentioned in the acknowledgments section? | Not Applicable | |

**Design**

| Study protocol | Information included in the manuscript? | In which section is the information available? (Reagents and Tools Table, Materials and Methods, Figures, Data Availability Section) |
|---|---|---|
| If study protocol has been **pre-registered, provide DOI in the manuscript**. For clinical trials, provide the trial registration number **OR** cite DOI. | Not Applicable | |
| Report the **clinical trial registration number** (at ClinicalTrials.gov or equivalent), where applicable. | Not Applicable | |

| Laboratory protocol | Information included in the manuscript? | In which section is the information available? (Reagents and Tools Table, Materials and Methods, Figures, Data Availability Section) |
|---|---|---|
| Provide DOI OR other citation details if **external detailed step-by-step protocols** are available. | Not Applicable | |

| Experimental study design and statistics | Information included in the manuscript? | In which section is the information available? (Reagents and Tools Table, Materials and Methods, Figures, Data Availability Section) |
|---|---|---|
| Include a statement about **sample size** estimate even if no statistical methods were used. | Yes | Materials and Methods |
| Were any steps taken to minimize the effects of subjective bias when allocating animals/samples to treatment (e.g. **randomization procedure**)? If yes, have they been described? | Not Applicable | |
| Include a statement about **blinding** even if no blinding was done. | Not Applicable | |
| Describe **inclusion/exclusion criteria** if samples or animals were excluded from the analysis. Were the criteria pre-established?<br><br>If sample or data points were omitted from analysis, report if this was due to attrition or intentional exclusion and provide justification. | Not Applicable | |
| For every figure, are **statistical tests** justified as appropriate? Do the data meet the assumptions of the tests (e.g., normal distribution)? Describe any methods used to assess it. Is there an estimate of variation within each group of data? Is the variance similar between the groups that are being statistically compared? | Yes | Materials and Methods |

| Sample definition and in-laboratory replication | Information included in the manuscript? | In which section is the information available? (Reagents and Tools Table, Materials and Methods, Figures, Data Availability Section) |
|---|---|---|
| In the figure legends: state number of times the experiment was **replicated** in laboratory. | Yes | Figures |
| In the figure legends: define whether data describe **technical or biological replicates**. | Yes | Figures |

## Ethics

| Ethics | Information included in the manuscript? | In which section is the information available? (Reagents and Tools Table, Materials and Methods, Figures, Data Availability Section) |
|---|---|---|
| Studies involving **human participants**: State details of **authority granting ethics approval** (IRB or equivalent committee(s), provide reference number for approval. | Yes | Materials and Methods |
| Studies involving **human participants**: Include a statement confirming that **informed consent** was obtained from all subjects and that the experiments conformed to the principles set out in the WMA Declaration of Helsinki and the Department of Health and Human Services Belmont Report. | Not Applicable | |
| Studies involving **human participants:** For publication of **patient photos**, include a statement confirming that consent to publish was obtained. | Not Applicable | |
| Studies involving experimental **animals**: State details of **authority granting ethics approval** (IRB or equivalent committee(s), provide reference number for approval. Include a statement of compliance with ethical regulations. | Yes | Materials and Methods |
| Studies involving **specimen and field samples:** State if relevant **permits** obtained, provide details of authority approving study; if none were required, explain why. | Not Applicable | |

| Dual Use Research of Concern (DURC) | Information included in the manuscript? | In which section is the information available? (Reagents and Tools Table, Materials and Methods, Figures, Data Availability Section) |
|---|---|---|
| Could your study fall under dual use research restrictions? Please check biosecurity documents and list of **select agents and toxins** (CDC): https://www.selectagents.gov/sat/list.htm | Not Applicable | |
| If you used a select agent, is the security level of the lab appropriate and reported in the manuscript? | Not Applicable | |
| If a study is subject to dual use research of concern regulations, is the name of the **authority granting approval and reference number** for the regulatory approval provided in the manuscript? | Not Applicable | |

## Reporting

The MDAR framework recommends adoption of discipline-specific guidelines, established and endorsed through community initiatives. Journals have their own policy about requiring specific guidelines and recommendations to complement MDAR.

| Adherence to community standards | Information included in the manuscript? | In which section is the information available? (Reagents and Tools Table, Materials and Methods, Figures, Data Availability Section) |
|---|---|---|
| State if relevant guidelines or checklists (e.g., **ICMJE, MIBBI, ARRIVE, PRISMA**) have been followed or provided. | Not Applicable | |
| For **tumor marker prognostic studies,** we recommend that you follow the **REMARK** reporting guidelines (see link list at top right). See author guidelines, under 'Reporting Guidelines'. Please confirm you have followed these guidelines. | Not Applicable | |
| For **phase II and III randomized controlled trials**, please refer to the **CONSORT** flow diagram (see link list at top right) and submit the CONSORT checklist (see link list at top right) with your submission. See author guidelines, under 'Reporting Guidelines'. Please confirm you have submitted this list. | Not Applicable | |

## Data Availability

| Data availability | Information included in the manuscript? | In which section is the information available? (Reagents and Tools Table, Materials and Methods, Figures, Data Availability Section) |
|---|---|---|
| Have **primary datasets** been deposited according to the journal's guidelines (see 'Data Deposition' section) and the respective accession numbers provided in the Data Availability Section? | Yes | Materials and Methods, Data Availability Section |
| Were **human clinical and genomic datasets** deposited in a public access-controlled repository in accordance to ethical obligations to the patients and to the applicable consent agreement? | Not Applicable | |
| Are **computational models** that are central and integral to a study available without restrictions in a machine-readable form? Were the relevant accession numbers or links provided? | Not Applicable | |
| If publicly available data were reused, provide the respective **data citations in the reference list**. | Not Applicable | |
